# Atomically Interfacial Engineering on Molybdenum Nitride Quantum Dots Decorated N‐doped Graphene for High‐Rate and Stable Alkaline Hydrogen Production

**DOI:** 10.1002/advs.202204949

**Published:** 2022-10-26

**Authors:** Yichao Huang, Wenbo Zhou, Weichao Kong, Lulu Chen, Xiaolong Lu, Hanqing Cai, Yongrui Yuan, Lianming Zhao, Yangyang Jiang, Haitao Li, Limin Wang, Lin Wang, Hang Wang, Jiangwei Zhang, Jing Gu, Zhuangjun Fan

**Affiliations:** ^1^ State Key Laboratory of Heavy Oil Processing School of Materials Science and Engineering China University of Petroleum Qingdao Shandong 266580 P. R. China; ^2^ College of Energy Material and Chemistry College of Chemistry and Chemical Engineering Inner Mongolia University Hohhot 010021 P. R. China; ^3^ Department of Chemistry and Biochemistry San Diego State University 5500 Campanile Drive San Diego CA 92182‐1030 USA

**Keywords:** electrocatalysis, hydrogen evolution reaction, molybdenum nitrides, polyoxometalates, quantum dots

## Abstract

The development of low‐cost, high‐efficiency, and stable electrocatalysts for hydrogen evolution reaction (HER) under alkaline conditions is a key challenge in water electrolysis. Here, an interfacial engineering strategy that is capable of simultaneously regulating nanoscale structure, electronic structure, and interfacial structure of Mo_2_N quantum dots decorated on conductive N‐doped graphene via codoping single‐atom Al and O (denoted as AlO@Mo_2_N‐NrGO) is reported. The conversion of Anderson polyoxometalates anion cluster ([AlMo_6_O_24_H_6_]^3−^, denoted as AlMo6) to Mo_2_N quantum dots not only result in the generation of more exposed active sites but also in situ codoping atomically dispersed Al and O, that can fine‐tune the electronic structure of Mo_2_N. It is also identified that the surface reconstruction of Al—OH hydrates in AlO@Mo_2_N quantum dots plays an essential role in enhancing hydrophilicity and lowering the energy barriers for water dissociation and hydrogen desorption, resulting in a remarkable alkaline HER performance, even better than the commercial 20% Pt/C. Moreover, the strong interfacial interaction (Mo—N bonds) between AlO@Mo_2_N and N‐doped graphene can significantly improve electron transfer efficiency and interfacial stability. As a result, outstanding stability over 300 h at a current density higher than 100 mA cm^−2^ is achieved, demonstrating great potential for the practical application of this catalyst.

## Graphical abstract

An atomically interfacialengineering strategy is developed for simultaneous regulation of nanostructure, electronic structure and interface on single atomic aluminum and oxygenco‐doped molybdenumnitride quantum dots decorated conductive nitrogen‐dopedgraphene (AlO@Mo_2_N‐NrGO), which shows a remarkable high‐rate alkaline HER performance, superior to commercial platinumcarbon (20% Pt/C), and an outstanding stability over 300 hours at 114 mA cm^−2^.

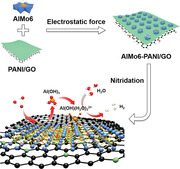

## 1 Introduction

Electrochemically producing “green hydrogen” via water splitting from renewable energy sources is an acknowledged path to accomplish the global mission of carbon neutrality.^[^ ^1^ ^]^ Nowadays, there are three main electrochemical water splitting technologies for hydrogen generation: i) alkaline water electrolysis,^[^ ^2^ ^]^ ii) proton exchange membrane water electrolysis,^[^ ^3^ ^]^ and iii) solid oxide water electrolysis.^[^ ^4^ ^]^ Among these water electrolysis technologies, the alkaline water electrolysis stands out to be more mature and economically feasible, since it has been commercialized for more than 100 years.^[^ ^2^, ^5^ ^]^ However, the considerably sluggish hydrogen evolution reaction (HER) kinetics in alkaline conditions, which is two to three orders of magnitude lower than that in acidic conditions,^[^ ^5b^ ^]^ has led to high‐energy consumption and hindered the advancement in alkaline water splitting technology.^[^ ^2^, ^6^ ^]^ To date, platinum (Pt)‐based compounds are proved to be the most efficient catalysts for alkaline HER, but the scarcity and high cost limit their large‐scale application.^[^ ^7^ ^]^ Therefore, developing a low‐cost, high‐efficiency, and stable non‐noble metal electrocatalyst for alkaline HER is highly desired.

Thanks to the efforts of many researchers, numerous noble‐metal‐free catalysts have been identified as potential candidates for alkaline HER, such as transition metal nitrides (TMNs),^[^ ^8^ ^]^ chalcogenides,^[^ ^5^, ^9^ ^]^ phosphides,^[^ ^10^ ^]^ carbides,^[^ ^11^ ^]^ and metal–organic frameworks (MOFs).^[^ ^12^ ^]^ Among these catalysts, TMNs has drawn considerable attention because of their unique electronic structures, high conductivity, superior chemical stability in a wide pH range and mechanical robustness.^[^ ^13^ ^]^ More importantly, the M—N bonding of TMNs modifies the nature of the d‐band of the parent metal, leading to a contraction of the metal d‐band and a higher density of states (DOS) of near the Fermi level, which is similar to those of group VIII noble metals.^[^ ^8^, ^14^ ^]^ More specifically, molybdenum nitride (Mo_2_N) has been intensively explored as electrocatalyst for HER.^[^ ^8a^ ^]^ Unfortunately, Mo_2_N exhibits moderate HER performance because of the limited amount of active sites and strong Mo—H bond, which induced sluggish kinetics for H_2_ formation.^[^ ^15^ ^]^ Pioneering studies have proposed that nanostructure engineering with tailored morphology and good dispersity can help to increase surface area, expose more active sites, facilitate mass transport, and shorten the electrolyte diffusion distance, thus, enhancing the HER performances.^[^ ^8^, ^16^ ^]^ For example, 2D Mo_2_N has been found to exhibit better HER activities than its bulk form.^[^ ^17^ ^]^ However, the synthesis of Mo_2_N commonly requires high temperature, which may cause pulverization and agglomeration during the nitridation of transition metal oxides/hydroxides.^[^ ^8a^ ^]^ Alternatively, electronic‐structure engineering, such as chemical doping,^[^ ^8^, ^18^ ^]^ defects,^[^ ^19^ ^]^ or hetero‐structures,^[^ ^8^, ^20^ ^]^ has also been proved to efficiently enhance HER activities. However, it is still a grand challenge to integrate electronic‐structure engineering with nanostructure engineering for achieving a high‐rate HER performance.

Polyoxometalates (POMs) are a kind of inorganic molecular clusters with small sizes (≈1 nm).^[^ ^21^ ^]^ Pioneering studies have employed POMs as structural well‐defined precursors for the synthesis of molybdenum (Mo) and tungsten (W)‐based electrocatalysts.^[^ ^12^, ^22^ ^]^ POMs clusters are electronegative, which can be efficiently anchored onto the surface of positively charge substrates, ensuring the uniform distribution of Mo (or W) source on substrates and the formation of small‐sized nanoparticles during high‐temperature nitridation.^[^ ^22^ ^]^ Furthermore, Anderson‐typed POMs are well‐defined 1:6 heteropolyanion clusters ([XMo_6_O_24_H_6_]*^n^* ^−^, denoted as XMo6) consisting of a single metal heteroatom XO6 octahedron (X =, e.g., a first‐row transition metal) with six edge‐sharing MoO6 octahedrons,^[^ ^21^, ^23^ ^]^ which can provide an opportunity for fine‐tune the electronic structure of Mo_2_N with precise atomic doping. Recently, we have reported using Anderson‐typed POMs clusters as the precursors to synthesize a series of ultrathin metallic 1T‐MoS_2_ nanosheets with well‐controlled atomic doping, which show outstanding alkaline HER activity.^[^ ^9^, ^24^ ^]^ However, to improve the electron transfer efficiency and catalytic stability, their interfacial interaction between the electrocatalysts and conductive substrates under long‐term electrocatalysis should be considered. Inspired by this work, we propose to contract ultrasmall Mo_2_N with precise heteroatom doping and strong covalent bonding onto a highly conductive carbon network to alleviate the aforementioned issues for Mo_2_N.

**Figure 1:**
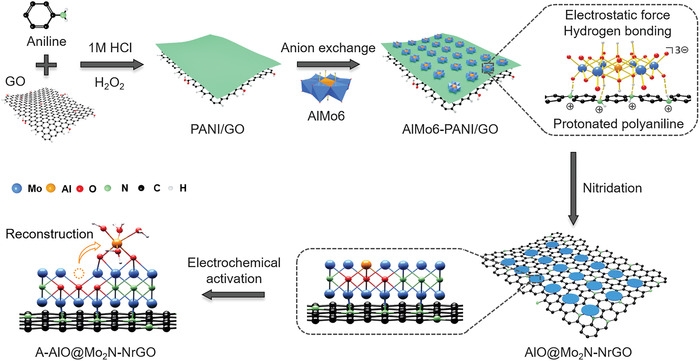
Schematic illustration of the synthesis of AlO@Mo_2_N‐NrGO quantum dots. The activated AlO@Mo_2_N‐NrGO (denoted as A‐AlO@Mo_2_N‐NrGO) structure with absorbed Al—O species derived from self‐optimization of AlO@Mo_2_N‐NrGO via electrochemical activation in alkaline electrolyte is also presented.

Here, we report an atomically interfacial engineering strategy for simultaneously regulating nanostructure, electronic structure, and interfacial structure of single‐atom Al and O codoped Mo_2_N quantum dots on conductive N‐doped graphene (AlO@Mo_2_N‐NrGO). The well‐defined Anderson‐typed POMs anion clusters ((NH_4_)_3_[AlMo_6_O_24_H_6_] (denoted as AlMo6) are anchored on the surface of protonated polyaniline/graphene oxides (PANI/GO) nanosheets via electrostatic and H bonding interactions (**Figure** **1**), which contribute to avoiding the agglomeration of Mo_2_N nanoparticles during the nitridation. Moreover, the Al and O sources derived from AlMo6 clusters can lead to in situ chemical doping in AlO@Mo_2_N at an atomic level, which can tune the electronic structure and expose more active sites. In addition, the strong interfacial Mo–N bonds between AlO@Mo_2_N and N‐doped graphene can significantly improve electron transfer efficiency and stability. Further experiments and theoretical calculations reveal that the surface reconstruction of Al—OH hydrates in AlO@Mo_2_N brings down energy barriers of both water dissociation and hydrogen desorption, leading to an effective self‐optimizing behavior and superior alkaline HER activity. As a result, the optimized AlO@Mo_2_N‐NrGO electrocatalyst produces an extremely low overpotential of 285 mV to achieve a high current density of 400 mA cm^−2^ for HER in a strong basic electrolyte (1.0 m KOH), superior to commercial 20% Pt/C and most of reported electrocatalysts. Moreover, outstanding stability over 300 h of continuous electrolysis process at a high current density of 114 mA cm^−2^ is achieved.

## 2 Results and Discussion

### 2.1 Synthesis and Characterization of AlO@Mo_2_N‐NrGO

**Figure 2:**
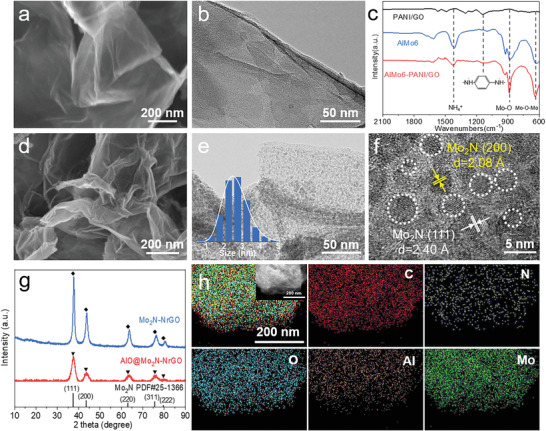
Structure characterizations of AlMo6‐PANI/GO and AlO@Mo_2_N‐NrGO. a–c) characterizations of AlMo6‐PANI/GO. a) SEM image. b) TEM image. c) FT‐IR spectra. d–g) characterizations of the AlO@Mo_2_N‐NrGO. d) SEM image. e) TEM image (inset: Particle size distribution). f) HRTEM image (the white dotted circles represent Mo_2_N quantum dots). g) PXRD patterns of AlO@Mo_2_N‐NrGO and Mo_2_N‐NrGO. h) EDX elemental mappings (inset: SEM image corresponding to EDX mappings).

The overall synthetic route for preparing AlO@Mo_2_N‐NrGO electrocatalyst is illustrated in Figure 1. First, an ultrathin protonated polyaniline (PANI) layer was coated on GO nanosheets according to our previous work with modification.^[^ ^25^ ^]^ Protonated PANI/GO nanosheet, which preserved the 2D nanosheets structure of pristine GO, was created (see Figure S1a,b, Supporting Information). Then, the Anderson‐typed POMs ((NH_4_)_3_[AlMo_6_O_24_H_6_], denoted as AlMo6) anion clusters were uniformly anchored on the surface of positively charged PANI/GO nanosheets by electrostatic interaction and hydrogen bonding, forming uniform AlMo6‐PANI/GO nanosheet structure, as confirmed by SEM (**Figure** **2**a; and Figure S1d, Supporting Information) and transmission electron microscopy (TEM, Figure 2b). Without the presence of PANI, the AlMo6 anion clusters could not uniformly disperse on the surface of GO nanosheets, instead, large agglomerations will be formed (Figure S1c, Supporting Information). Compared to pure AlMo6, the NH_4_^+^ peak at 1405 cm^−1^ was significantly decreased in AlMo6‐PANI/GO, indicating some NH_4_^+^ cations were replaced by the protonated PANI/GO (Figure 2c). The characteristic peaks of AlMo6 cluster located at 923 cm^−1^ (Mo=O), 879 cm^−1^ (Mo—O—Mo), and 635 cm^−1^ (Mo—O—Mo) were well retained in AlMo6‐PANI/GO (Figure 2c). Moreover, the uniform distribution of Al, Mo, C, N, and O elements in AlMo6‐PANI/GO was illustrated by energy dispersive X‐ray (EDX) elemental mappings, as shown in Figure S2 (Supporting Information). All these results indicate the uniform distribution of AlMo6 clusters on the surface of PANI/GO.

In the subsequent nitridation process, urea was exploited as a nitrogen source. After the nitridation process under hydrogen/argon atmosphere at 700 °C for 3 h, the AlMo6‐PANI/GO precursor was converted into the targeted AlO@Mo_2_N‐NrGO electrocatalyst (see the Experimental Section for more details). The nitridation temperature has also been investigated, which shows the optimized nitridation temperature is 700 °C, and thus AlO@Mo_2_N‐NrGO‐700 is named AlO@Mo_2_N‐NrGO for short in this work (Figure S3, Supporting Information). The nanosheet structure of AlMo6‐PANI/GO is well retained in AlO@Mo_2_N‐NrGO, as shown in Figure 2d. More detailed microstructure analysis of AlO@Mo_2_N‐NrGO via TEM imaging (Figure 2e) shows the presence of numerous ultrasmall quantum dots, embedded on the NrGO nanosheets, with an average diameter of 2.5 nm (inset in Figure 2e). This result confirms the uniform dispersion of AlMo6 clusters on PANI/GO nanosheets contributes to avoiding the agglomeration of AlO@Mo_2_N quantum dots on N‐doped graphene substrates (NrGO).

The high‐resolution TEM (HRTEM) image clearly discloses that the AlO@Mo_2_N‐NrGO electrocatalyst composes well‐crystallized quantum dots decorated on an amorphous carbon matrix (Figure 2f). The apparent lattice fringes on the nanoparticles with distances of 2.4 and 2.08 Å can be attributed to (1 1 1) and (2 0 0) crystallographic planes of Mo_2_N, respectively, in agreement with the powder X‐ray diffraction (PXRD) results (Figure 2g). As a control, the Mo_2_N supported on nitrogen‐doped graphene (defined as Mo_2_N‐NrGO) has also been synthesized using Mo7‐PANI/GO as a precursor. Both AlO@Mo_2_N‐NrGO and Mo_2_N‐NrGO exhibit clear diffraction peaks at 2*θ* = 37.37°, 43.45°, 63.11°, 75.73°, and 79.71°, corresponding to the (111), (200), (220), (311), and (222) crystal planes of the cubic Mo_2_N (PDF#25‐1366), respectively. It should be noted that the diffraction peaks of AlO@Mo_2_N‐NrGO are wider and lower than those of Mo_2_N‐NrGO, indicating the doping of Al atoms can effectively prevent the agglomeration of Mo_2_N nanoparticles (Figure 2g). Moreover, the uniform distribution of C, N, O, Al, and Mo elements in AlO@Mo_2_N‐NrGO is illustrated by the EDX elemental mapping (Figure 2h). The atomic ratios of Al/Mo (1.00:4.20) and N/Mo (1.00:1.96) in AlO@Mo_2_N‐NrGO are confirmed by the inductively coupled plasma‐atomic emission spectrometry (ICP‐AES) and EDX (see Table S4, Supporting Information).

**Figure 3:**
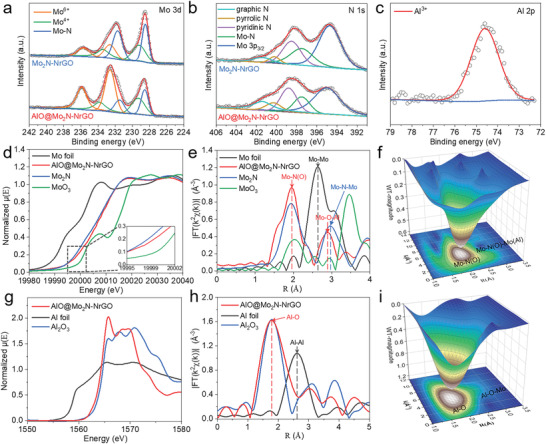
XPS and XAS analysis. a–c) High‐resolution XPS signals of a) Mo 3d, b) N 1s, c) Al 2p for AlO@Mo_2_N‐NrGO and Mo_2_N‐NrGO. d) Normalized Mo K‐edge X‐ray absorption near edge structure (XANES) spectra of AlO@Mo_2_N‐NrGO and reference samples (commercial Mo foil, Mo_2_N, and MoO_3_). e) The corresponding k^2^‐weighted FT profiles in R‐space from extended X‐ray absorption fine structure (EXAFS) at Mo K‐edge collected on AlO@Mo_2_N‐NrGO and reference samples. f) 3D contour wavelet transform (WT) representation with 2D projection of Mo K‐edge EXAFS for AlO@Mo_2_N‐NrGO. g) Normalized Al K‐edge XANES spectra of AlO@Mo_2_N‐NrGO and reference samples (commercial Al foil and Al_2_O_3_). h) The corresponding k^2^‐weighted FT profiles in R‐space from EXAFS at Al K‐edge collected on AlO@Mo_2_N‐NrGO and reference samples. i) 3D contour WT representation with 2D projection of Al K‐edge EXAFS for AlO@Mo_2_N‐NrGO.

To evaluate the chemical composition and valence state of electrocatalysts, the AlO@Mo_2_N‐NrGO nanosheet is further studied by X‐ray photoelectron spectroscopy (XPS). As shown in Figure S4a (Supporting Information), the XPS spectra of AlO@Mo_2_N‐NrGO indicate the presence of C, N, O, Al, and Mo. The high‐resolution Mo 3d spectrum of AlO@Mo_2_N‐NrGO is deconvoluted into three pairs of peaks (**Figure** **3**a). The peaks at the binding energies of 228.5 and 231.8 eV can be ascribed to Mo 3d 5/2 and Mo 3d 3/2 of Mo—N in Mo_2_N, respectively. The peaks located at higher binding energy correspond to Mo^4+^ (229.2 and 233.4 eV) and Mo^6+^ (232.6 and 235.7 eV), which might be originated from the surface oxidation.^[^ ^11^, ^20^, ^26^ ^]^ Interestingly, the Mo^6+^ peak intensity of AlO@Mo_2_N‐NrGO is much higher than that of Mo_2_N‐NrGO, indicating that doping Al increases surface oxidation degree of the Mo_2_N species. Besides, the N 1s spectrum of AlO@Mo_2_N‐NrGO is deconstructed into five peaks at 395.1, 397.5, 398.7, 400.4, and 401.4 eV, corresponding to the existence of Mo 3p_3/2_, Mo—N, pyridinic‐N, pyrrolic‐N, and graphic‐N, respectively (Figure 3b).^[^ ^20^, ^26^ ^]^ Further, the existence of Mo—N bond (397.4 eV) was confirmed by XPS, indicating that Mo_2_N quantum dots can be strongly anchored on the NrGO via Mo—N bond.^[^ ^25^ ^]^ The Mo—N binding energy of AlO@Mo_2_N‐NrGO is slightly lower than that of Mo_2_N‐NrGO due to the formation of Al—O—Mo species as shown by the Al 2p spectrum (Figure 3c).^[^ ^27^ ^]^ The main peak of C 1s spectrum located at 284.6 eV can be attributed to the existence of graphite carbon matrix (Figure S4b, Supporting Information), which corresponds well with the Raman (Figure S5, Supporting Information) and thermal gravimetric analysis (Figure S6, Supporting Information) results.^[^ ^28^ ^]^ Specifically, the peak at 285.5 eV can be ascribed to the formation of C—N bonds, indicating that the graphene is nitrided during nitridation. The O 1s spectrum is deconstructed into two peaks at 530.7 eV and 532.3 eV, assigned to O—Mo and O—C bonds, respectively (Figure S4c, Supporting Information).^[^ ^29^ ^]^ Moreover, the porous property of AlO@Mo_2_N‐NrGO nanosheet can be revealed by the N_2_ adsorption–desorption isotherm measurements (Figure S7, Supporting Information).

To make clear the effects of Al doping on the atomic coordination structure and electronic structure, the X‐ray absorption spectroscopy (XAS) analyses of both Mo K‐edge and Al K‐edge are conducted (Figure 3d–i; and Figures S8 and S9, Supporting Information). Figure 3d shows that the Mo K‐edge XANES of AlO@Mo_2_N‐NrGO resembles that of pure Mo_2_N, suggesting very similar local coordination environments around the Mo center for AlO@Mo_2_N‐NrGO and Mo_2_N, consistent with XRD results. However, the absorption edge for AlO@Mo_2_N‐NrGO shifts to a higher position as compared with that of pure Mo_2_N, while it is lower than those of MoO_3_, suggesting partial oxidation of Mo atoms in AlO@Mo_2_N‐NrGO, in agreement with XPS results (Figure 3d). As shown in the Fourier transformed (FT) R‐space from Mo K‐edge extended X‐ray absorption fine structure (EXAFS) of AlO@Mo_2_N‐NrGO, in comparison with Mo foil, Mo_2_N, and MoO_3_ samples (Figure 3e), the first‐shell interaction peaks of AlO@Mo_2_N‐NrGO located at 1.96 Å, which is between first peaks of Mo_2_N (1.93 Å) and MoO_3_ (2.02 Å), indicating the coexistence of Mo—N and Mo—O bonds in AlO@Mo_2_N‐NrGO. As for the second‐shell interaction, the peak of AlO@Mo_2_N‐NrGO (2.91 Å) is slightly lower than that of Mo_2_N (2.97 Å, Mo—N—Mo), which may be resulted from the hybridization of Mo—O—Al bond via Al doping. Moreover, the 3D contour wavelet transform (WT) representation of the EXAFS signal (Figure 3f; and Figure S8, Supporting Information) shows that in the coordinate system composed of k and R space, the Mo—N—Mo bonds in pure Mo_2_N are located at ≈(2.04, 2.97). At the same time, Mo—O—Mo bond in MoO_3_ is located at ≈(2.87, 3.46), and the Mo—N(O) —Mo(Al) bond in AlO@Mo_2_N‐NrGO is located at ≈(2.23, 2.91). All the above results further confirm the presence of both Mo—N—Mo and Mo—O—Al in AlO@Mo_2_N‐NrGO (Figure 3f; and Figure S8, Supporting Information).

In addition to the Mo edge, the XAS spectrum of AlO@Mo_2_N‐NrGO at the Al K‐edge is measured to examine its local structure. As shown in Figure 3g, the Al K‐edge XANES of AlO@Mo_2_N‐NrGO is much higher than that of Al foil but is similar to that of commercial Al_2_O_3_, indicating similar trivalence of the Al element, in agreement with XPS (Figure 3c). However, the absorption edge of Al K‐edge for AlO@Mo_2_N‐NrGO shifts to a lower position as compared to that of pure Al_2_O_3_, suggesting a partial reduction of the Al—O species. This observation is reasonable since these Al—O species are doped into the reductive Mo_2_N phase (Figures 2g and 3g). In addition, one obvious peak at around 1.78 Å in the first coordination shell can be observed from the radial distance space spectra *χ*(R) (Figure 3h). Different from the peak position of Al—Al bond (2.61 Å) in Al foil, the scattering path position in the first coordination shell of AlO@Mo_2_N‐NrGO is consistent with the first coordination shell of Al_2_O_3_, indicating the existence of Al—O bonding similar to Al_2_O_3_. The 3D contour WT representation of the Al K‐edge EXAFS signal shows that the Al—O and Al—O—Al bonds in pure Al_2_O_3_ are located at ≈(5.2, 1.82) and ≈(6.2, 2.82), respectively, while the Al—O and Mo—O—Al bond in AlO@Mo_2_N‐NrGO are located at ≈(5.0, 1.84) and ≈(8.2, 2.84) (Figure 3i; and Figure S9, Supporting Information).

All these results suggest that the AlO@Mo_2_N quantum dots with atomically dispersed Al and O atoms has been obtained. Later, it demonstrates those dopants facilitate the exposure of more active sites and fine‐tune chemical environments of Mo_2_N to achieve a superior alkaline HER performance.

### 2.2 Evaluation of Electrochemical HER Performance

**Figure 4:**
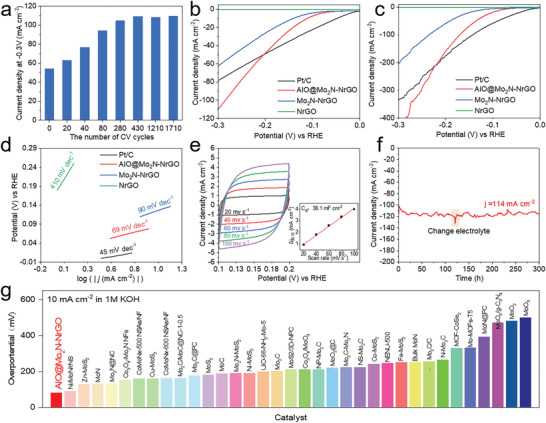
HER performances of electrocatalysts. a) Current density at −0.3 V of AlO@Mo_2_N‐NrGO after different CV cycles in 1.0 m KOH with a scan rate of 5 mV s^−1^ under a three‐electrode configuration. b,c) Polarization curves of Pt/C, AlO@Mo_2_N‐NrGO, Mo_2_N‐NrGO, and NrGO b) without and c) with IR compensation. d) Tafel plots. e) CVs of AlO@Mo_2_N‐NrGO with different rates from 20 to 100 mV s^−1^. Inset: the capacitive current at 0.15 V as a function of different scan rates for the AlO@Mo_2_N‐NrGO. f) The long‐term durability tests at *η* = 300 mV for 300 h. g) Comparison of HER performances in 1.0 m KOH for AlO@Mo_2_N‐NrGO with other Mo‐based electrocatalysts.

A self‐optimizing HER activity for AlO@Mo_2_N‐NrGO electrocatalyst (see **Figure** **4**a) was noticed in the cyclic voltammetry (CV) cycles. The current density corresponding to −0.3 V versus reversible hydrogen electrode (RHE) increases from 54.12 to 109.10 mA cm^−1^ after 430 CV cycles and holds steady from 430 to 1710 CV cycles, while this self‐optimizing phenomenon is observed in Mo_2_N‐NrGO (Figure S10, Supporting Information). As shown in Figure 4b, the activated AlO@Mo_2_N‐NrGO electrocatalyst exhibits a better HER activity with an overpotential of *η*_10_ = 111 mV (without IR compensation), compared to Mo_2_N‐NrGO (*η*_10_ = 141 mV), while the NrGO has almost no HER activity. For clarity, the following discussion of HER performance of AlO@Mo_2_N‐NrGO is all based on the CV‐activated sample. The HER activity of AlO@Mo_2_N‐NrGO is even superior to that of commercial 20% Pt/C at overpotentials (*η* > 203 mV). After IR compensation, the AlO@Mo_2_N‐NrGO still maintains the best performance, which outperforms Pt/C at an overpotential *η* > 203 mV (see Figure 4c). Moreover, only a small overpotential of 285 mV can generate a high current density of 400 mA cm^−2^, superior to most reported high‐current‐density electrocatalysts. Additionally, the mass activity of AlO@Mo_2_N‐NrGO is about twice better than the Mo_2_N‐NrGO. This is demonstrated by achieving a high current density of 1000 mA mg^−1^ at a small overpotential of 275 mV (see Figure S11, Supporting Information). Therefore, the low cost and highly efficient AlO@Mo_2_N‐NrGO electrocatalyst show great potential for commercialized alkaline water electrolysis application that requires a high current density (≈400–600 mA cm^−2^).^[^ ^30^ ^]^

The HER kinetics of electrocatalysts are further evaluated using Tafel plots as shown in Figure 4d. The Tafel slope of AlO@Mo_2_N‐NrGO (64 mV dec^−1^) is slightly higher than that of 20% Pt/C (45 mV dec^−1^) but much lower than those of the references, indicating AlO@Mo_2_N‐NrGO electrocatalyst proceeds via a Volmer–Heyrovsky mechanism, where desorption of hydrogen is the rate‐limiting step.^[^ ^31^ ^]^ The exchange current density (*j*_0_) derived from the Tafel equation is used to evaluate the intrinsic HER activity (Table S5, Supporting Information). The results show that the exchange current density of AlO@Mo_2_N‐NrGO (0.61 mA cm^−2^) is very close to that of 20% Pt/C (0.95 mA cm^−2^), indicating its high intrinsic HER activity. Electrochemical impedance spectroscopy (EIS) of AlO@Mo_2_N‐NrGO at various overpotentials shows similar impedance properties, suggesting similar electrochemical processes proceed in 1.0 m KOH at these overpotentials (Figure S12, Supporting Information). As well, EIS for AlO@Mo_2_N‐NrGO, Mo_2_N‐NrGO and NrGO performed at open circuit potentials are measured (Figure S13, Supporting Information). Herein, results are fitted by a simplified equivalent circuit (inset in Figure S13, Supporting Information). The optimized AlO@Mo_2_N‐NrGO shows a much lower charge‐transfer resistance (*R*_ct_) in comparison with that of Mo_2_N‐NrGO (Table S5, Supporting Information), suggesting improved charge‐transfer properties and HER kinetics. The electrochemical active surface area (ECSA) can shed light on different electrochemically active sites, while electrochemical double‐layer capacitances (*C*_dl_) are proportional to the ECSA. *C*_dl_ via different scan rates of CVs were measured to assess the ECSA. CV is performed in a range from 0.1 to 0.2 V at rates varying from 20 to 100 mv s^−1^. The *C*_dl_ of AlO@Mo_2_N‐NrGO is 38.1 mF cm^−2^, which is much higher than that of Mo_2_N‐NrGO (13.8 mF cm^−2^) and NrGO (6.03 mF cm^−2^) (see Figure 4e; and Figure S14, Supporting Information). These results demonstrate that AlO@Mo_2_N‐NrGO presents a significantly enhanced HER activity compared to the Mo_2_N‐NrGO, which shows that doping Al and O atoms into Mo_2_N excel the HER activity.

Long‐term stability under a high current density is also a critical factor that rules the performance of alkaline water electrolysis. Chronoamperometry (CA) analysis reveals a stable current density for 300 h at a high current density of 114 mA cm^−2^, where 99.3% of its initial current density was retained (Figure 4f). After 300 h of electrolysis, the XRD pattern of AlO@Mo_2_N‐NrGO reveals that the AlO@Mo_2_N‐NrGO retains its initial crystal structure integrity, further confirming its superior chemical stability in strong alkaline solution (Figure S15, Supporting Information). Comparing the overpotentials (*η*_10_) in alkaline conditions with the most recently reported Mo‐based HER electrocatalysts (Figure 4g; and Table S6, Supporting Information), the as‐prepared AlO@Mo_2_N‐NrGO electrocatalyst definitely stands out as one of the best. The turnover frequency (TOF) of the AlO@Mo_2_N‐NrGO is also calculated to estimate its intrinsic catalytic activity. The AlO@Mo_2_N‐NrGO has a higher TOF value (0.7 s^−1^ at an overpotential of 300 mV than that of the Mo_2_N‐NrGO (0.3 s^−1^) (Figure S16, Supporting Information). The Faradaic efficiency (FE) of AlO@Mo_2_N‐NrGO for H_2_ generation in 1.0 m KOH is measured to be ≈99.78% consistent with its theoretical value (Figure S17, Supporting Information). Moreover, the techno‐economic analysis result demonstrates that the cost of unit hydrogen (COUH) by AWE using AlO@Mo_2_N‐NrGO as an HER catalyst is only 0.68 US dollars per Nm^3^H_2_, which can serve 3.49 US dollars per Nm^3^H_2_ compared to commercial Pt/C (20 wt%) catalyst (details see Tables S1–S3, Supporting Information). All the above results prove that the as‐prepared AlO@Mo_2_N‐NrGO electrocatalyst has excellent alkaline HER activity and stability, demonstrating great potential for the practical application of this catalyst.

### 2.3 Structure–Activity Relationship

**Figure 5:**
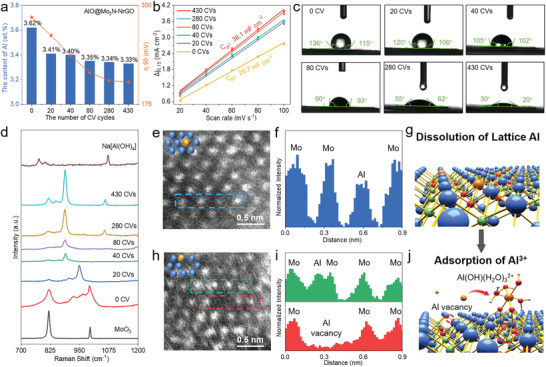
The study of self‐optimizing mechanism of AlO@Mo_2_N‐NrGO electrocatalyst. a) The contents of Al element and corresponding overpotentials at current density of 50 mA cm^−2^ (*η*_50_) with increasing CV cycles. b) The corresponding *C*_dl_ values with increasing CV cycles. c) Hydrophilic property increases with the increased CV cycles. d) Raman spectra of AlO@Mo_2_N‐NrGO after different numbers of CV. MoO_3_ and Na[Al(OH)_4_] were added as references. e) Aberration‐corrected atomic resolution High‐angle annular dark‐field scanning TEM (HAADF‐STEM) image of AlO@Mo_2_N‐NrGO without CV activation, the inset shows schematic diagram of the top‐view structure of AlO@Mo_2_N‐NrGO. f) Intensity profiles along the blue dotted box indicated in image e). g) Schematic diagram of AlO@Mo_2_N‐NrGO without CVs activation. h) Aberration‐corrected atomic resolution HAADF‐STEM image of A‐AlO@Mo_2_N‐NrGO after CV activation, the inset shows schematic diagram of the top‐view structure of A‐AlO@Mo_2_N‐NrGO. i) Intensity profiles along the green and red dotted box indicated in image i). j) Schematic diagram of A‐AlO@Mo_2_N‐NrGO after CV activation.

In order to understand the self‐optimizing mechanism of AlO@Mo_2_N‐NrGO electrocatalyst during the electrochemical activation process, factors, such as Al leaching, ECSA and hydrophilic property, have been investigated as the activation proceeds (**Figure** **5**).

As shown in Figure 5a, the percentage of Al in AlO@Mo_2_N‐NrGO decreases from 3.62% to 3.35% after 80‐cycles of CV, indicating some Al atoms may be leached into the 1.0 m KOH electrolyte. This result is expected to generate more HER active sites as confirmed by the increased *C*_dl_ values (Figure 5b; and Figure S18, Supporting Information). Herein, we postulate that Al atoms in AlO@Mo_2_N‐NrGO will react with OH^−^ to form Al—OH hydrates, which will consequently adsorb onto the surface of AlO@Mo_2_N‐NrGO to enhance the catalyst's hydrophilicity. As it has been observed that the contact angle of AlO@Mo_2_N‐NrGO decreases from 115° (a hydrophobic surface) to 20° (a hydrophilic surface) after 430 CV cycles (Figure 5c). Interestingly, the Al content was maintained at around 3.33% even after 430 CV cycles, while the HER activity keeps increasing with overpotential (*η*_50_) decreases from 290 to 203 mV after 430 CV cycles (Figure 5a), indicating the dissolution and adsorption of Al—OH hydrates on the surface of AlO@Mo_2_N‐NrGO may approach a saturation point during the electrochemical activation. The above evidence indicates that self‐optimizing HER performance of AlO@Mo_2_N‐NrGO can be attributed to the reconstruction of Al—OH hydrates on the surface of AlO@Mo_2_N‐NrGO during the electrochemical activation, which increases both the ECSA and hydrophilicity.

Raman spectra are further utilized to reveal the changes of chemical environment on the surface of AlO@Mo_2_N‐NrGO electrocatalyst (Figure 5d). Initially, the AlO@Mo_2_N‐NrGO shows two obvious bands at ≈≈820 and 993 cm^−1^ (red curve in Figure 5d), similar to those of the reference MoO_3_ (black curve in Figure 5d), which can be attributed to the characteristic Mo—O—Mo and Mo=O vibrations of MoO_3_, respectively,^[^ ^32^ ^]^ indicating the surface of AlO@Mo_2_N‐NrGO is oxidized and covered with amorphous MoO_3_ species, in agreement with XPS and XAS results (Figure 3a,e). After 20 CV cycles, the Mo—O peaks disappear and a transition peak from Mo—O to Mo—N appears at 948 cm^−1^, indicating the amorphous MoO_3_ on the surface of AlO@Mo_2_N‐NrGO was completely dissolved. Further, after 40 CV cycles, characteristic Mo—N peaks at ≈817 and 849 cm^−1^ begin to emerge.^[^ ^33^ ^]^ Meanwhile, another visible band at around 1059 cm^−1^ that belongs to Al—O vibration of aluminates (Na[Al(OH)_4_]) can be seen after 280 CV cycles, indicating the Al dopants in the lattice of Mo_2_N can be dissolved out to form aluminates, such as Al(OH)(H_2_O)_3_^2+^, and adsorbed on the surface of AlO@Mo_2_N‐NrGO, leading to an increased ECSA and hydrophilicity.

To visually understand the self‐optimizing process of AlO@Mo_2_N‐NrGO electrocatalyst, the high‐angle annular dark‐field scanning TEM (HAADF‐STEM) analysis is further performed. As shown in Figure 5e, AlO@Mo_2_N‐NrGO exhibits an obvious intensity variation of Al and Mo atoms, as confirmed by the intensity profiles of Figure 5f, indicating that Al atoms are successfully doped into the Mo_2_N lattice in the pristine AlO@Mo_2_N‐NrGO (Figure 5g), which may be attacked by OH^−^ during the CV activation in the alkaline electrolyte and gradually dissolved to form Al—OH species in the alkaline electrolyte, creating Al vacancies. Such a hypothesis has been proved by HAADF‐STEM result in Figure 5h, where the AlO@Mo_2_N‐NrGO shows atomic vacancies after Al atoms were dissolved. Moreover, the dissolved aluminates may be adsorbed on the surface of Mo_2_N via the Mo—O—Al bond (Figure 5i), as confirmed by the EDX elemental mapping (Figure S19, Supporting Information) and the intensity profiles in Figure 5j, which shows enhanced bright spot around the position of Al vacancy. In conclusion, the self‐optimizing HER activity of AlO@Mo_2_N‐NrGO originated from the following two reasons: First, the initial formed amorphous Mo oxides on the surface of AlO@Mo_2_N‐NrGO are dissolved to expose more AlO@Mo_2_N active sites; Second, Al dopants are dissolved and react with electrolyte to form Al—OH species, which can be adsorbed on the surface of the AlO@Mo_2_N‐NrGO during CV activation, leading to an enhanced HER performance.

### 2.4 Density Functional Theory (DFT) Calculations

**Figure 6:**
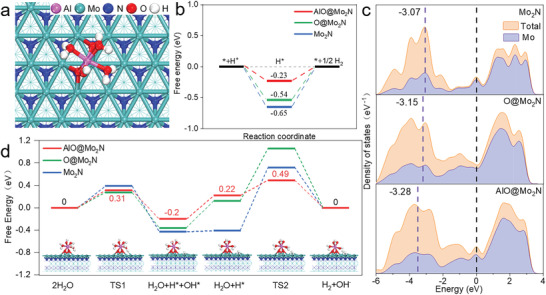
First‐principles calculations of the doping effect on alkaline HER performance. a) The monolayer structure of AlO@Mo_2_N. b) Hydrogen adsorption free energy diagram for AlO@Mo_2_N, O@Mo_2_N, and Mo_2_N. c) The DOSs of AlO@Mo_2_N, O@Mo_2_N, and Mo_2_N. The Fermi level marked by black dashed lines is set as energy zero. The d‐band centers of Mo are shown by purple dashed lines. d) Free energy diagrams of HER on the surface of different catalysts in alkaline solution.

Further insight into the underlying mechanism of the enhanced HER performance is deduced by first principles DFT calculation.^[^ ^20a^ ^]^ Theoretically, the alkaline HER pathway usually consists of four steps: the adsorption of H_2_O on catalyst, H_2_O dissociation step (Volmer), intermediate H* formation, and finally, H_2_ generation step (Heyrovsky or Tafel). The HER process involves the adsorption of H*, so the free energy of the adsorption of H*(Δ*G*_H*_) play a key role in HER. We calculate the Δ*G*_H*_ on the surfaces of activated AlO@Mo_2_N‐NrGO (a model that is adsorbed by Al—OH hydrates), O@Mo_2_N‐NrGO (a model that is with surface oxidation but without Al—OH hydrates) and Mo_2_N‐NrGO catalysts (a model that removes surface oxidation and Al—OH hydrates). The optimized catalyst models of AlO@Mo_2_N, O@Mo_2_N, and Mo_2_N are shown in **Figure** **6**a; and Figure S20 (Supporting Information). According to the experimental results, the reaction pathway of Volmer–Heyrovsky mechanism for alkaline HER has been confirmed (Figure 4d). It is generally believed that the free energy of adsorbed H (∆*G*_H*_) is a key decipher for predicting HER activity where an ideal ∆*G*_H*_ should be thermoneutral. Compared with Mo_2_N (−0.65) and O@Mo_2_N (−0.54), the ∆*G*_H*_ value of AlO@Mo_2_N (−0.23) is closer to the thermoneutral value (Figure 6b), demonstrating that surface adsorption of aluminates can effectively reduce the HER energetic barrier. It is worth noting that the total DOS of AlO@Mo_2_N, O@Mo_2_N, and Mo_2_N near the Fermi level is continuous without a bandgap, proving the metallic nature of AlO@Mo_2_N, O@Mo_2_N, and Mo_2_N. Figure 6c also shows the d‐band energetics of Mo for AlO@Mo_2_N, O@Mo_2_N, and Mo_2_N, with d‐band centers at −3.28, −3.15, and −3.07 eV, respectively. The left shift of d‐band of AlO@Mo_2_N away from the Fermi level indicates that the catalyst has a lower adsorption energy for H, which is beneficial for the desorption of H_2_.

A H_2_O molecule on top (Mo) site of Mo_2_N (111) directly decomposes into H* and OH* at two adjacent hollow sites via the initial O—H bond scission (i.e., Volmer reaction). This step is exothermic (−0.43 eV) with a free energy barrier of 0.39 eV (Figures S21 and S22, Supporting Information). Then, the adsorbed H* combines with a H in H_2_O to yield a H_2_ molecule and a hydroxyl species (i.e., Heyrovsky reaction). However, the Heyrovsky reaction is hindered by a high free energy barrier of 1.13 eV, suggesting low HER activity of Mo_2_N. In contrast, when the Mo_2_N surface is doped by O, the electronegative O atoms afford an efficient active site for adsorbing H*. For O@Mo_2_N, the free energy barriers of the Volmer and Heyrovsky steps are decreased to 0.27 and 0.94 eV (see Figures S21 and S23, Supporting Information), respectively, indicating an improved HER activity. This situation would be attributed to a higher nucleophilicity of the surface O atoms. Consistent with Mo_2_N, the HER on O@Mo_2_N is limited by the Heyrovsky step. Once Al—OH hydrates are absorbed on AlO@Mo_2_N, one of the coordinated waters in (H_2_O)_3_AlO_2_(OH) can be activated and decomposed into H* and OH* (Figure S24, Supporting Information). In this process, H atom on the activated H_2_O departs from the O atom and arrives at the hollow site of Mo_2_N(111), whereas the OH group is still stably coordinated with the Al ion, forming (H_2_O)_3_AlO_2_(OH)_2_. This reaction is feasible, since it is exothermic (0.2 eV) with a small energy barrier of 0.31 eV. Interestingly, the (H_2_O)_2_AlO_2_(OH)_2_ complex with a high oxidation state is very reactive and readily accepts a hydrogen of the water, forming (H_2_O)_3_AlO_2_(OH), resulting in a much‐lowered activation energy of 0.21 eV (Figures S21 and S25, Supporting Information). Subsequently, the H_2_ molecule is formed via H* adsorbed at Mo_2_N combining with another H coordinated with H_2_O in (H_2_O)_3_AlO_2_(OH). The free energy barrier (0.27 eV) of this Heyrovsky reaction is 0.04 eV lower than that of the former Volmer reaction (0.31 eV), suggesting that rate‐determining step of the HER is changed from the Heyrovsky step on Mo_2_N and O@Mo_2_N to the Volmer step on AlO@Mo_2_N. Furthermore, the calculated Gibbs free energy diagrams of HER are given in Figure 6d. The free energy barrier of the HER rate‐determining step is calculated to be 0.31, 0.94, and 1.13 eV for AlO@Mo_2_N, O@Mo_2_N, and Mo_2_N, respectively, suggesting that the HER activity increases following the order of Mo_2_N < O@Mo_2_N < AlO@Mo_2_N. All these results indicate that the adsorption of aluminates on the surface of AlO@Mo_2_N via the Mo—O—Al bonding can greatly enhance the electrocatalytic alkaline HER performance, in agreement with experimental results.

## 3 Conclusion

In summary, we report an atomically interfacial engineering strategy for simultaneous regulation of nanostructure, electronic structure, and interface on AlO@Mo_2_N‐NrGO. The uniformly anchored POMs anion clusters on the PANI/GO via strong electrostatic interaction and hydrogen bonding can effectively prevent the aggregation of Mo_2_N quantum dots. Moreover, the in situ codoping Al and O in Mo_2_N quantum dots can be achieved by using AlMo6 anion clusters as precursors, which can effectively tune the electronic structures of Mo_2_N. The leaching and reconstruction of Al—OH hydrates on the surface of AlO@Mo_2_N‐NrGO facilitate the exposure of more active sites and increase the surface hydrophilicity, leading to a self‐optimizing HER activity. The as‐optimized AlO@Mo_2_N‐NrGO electrocatalyst exhibits a remarkable alkaline HER performance with a low overpotential of 82 mV versus RHE at 10 mA cm^−2^, superior to the commercial 20% Pt/C at an overpotential larger than 203 mV versus RHE. Moreover, owing to strong interfacial interactions between AlO@Mo_2_N quantum dots and NrGO, outstanding stability over 300 h at a high current density of 114 mA cm^−2^ can be achieved. The experimental and theoretical results show that the reconstruction of Al—OH hydrates on the surface of AlO@Mo_2_N can effectively reduce the reaction energy barrier required for the Heyrovsky step and promote the desorption of H_2_ on the catalyst surface. Given the earth‐abundance and structural versatility of POMs, the molecular design of more POMs with different heteroatoms will allow the independent chemical tuning and optimization of electrocatalysts. This study can serve as a blueprint for low‐cost, high‐rate, and stable electrocatalysts for renewable H_2_ generation.

## 4 Experimental Section

### Materials and Characterizations

Aniline (C_6_H_7_N, ACS ≥99.0%) and Aluminum nitrate nonahydrate (Al(NO_3_)_3_·9H_2_O, AR 99.0%) were purchased from Aladdin Reagent Ltd. Hydrochloric acid (AR), hydrogen peroxide 30% aqueous solution (H_2_O_2_, AR), isopropyl alcohol (HPLC ≥99.7%), and urea (AR) were purchased from Sinopharm Chemical Reagent Co., Ltd. Ammonium heptamolybdate tetrahydrate (H_24_Mo_7_N_6_O_24_·4H_2_O, AR 99%) was purchased from Shanghai McLean Biochemical Technology Co., Ltd. Platinum carbon catalyst (20% Pt/C, HPT020), Nafion perfluorinated resin solution and Hydrophilic carbon paper were purchased from Shanghai Hesen Electric Co., Ltd. IR spectrum was measured by using ATR test bench and recorded on a SHIMADZU IRTracer‐100 FE‐IR spectrometer. Elemental analyses of Mo, Al were performed by ICP‐AES on Agilent 720ES Inductively Coupled Plasma Emission Spectrometer. Powder X‐ray diffraction characterization was performed on a Panako PANalytical X‐ray Diffractometer using Cu‐Ka radiation (*λ* = 1.5418 Å). The morphology and size of the nanostructured materials were characterized by an FEI Technai G2 F20 TEM. Scanning electron microscopy (SEM) was conducted on an FEI Nova nanoSEM 450. Elemental mappings on the material surface were performed on EDAX Phoenix Spectrometer. X‐ray photoelectron spectroscopy (XPS) experiments were carried out on a Thermo fisher Escalab 250Xi with monochromated Al K*α* radiation, calibrated with C 1s = 284.8 eV. Raman spectra were recorded using a Renishaw Qontor confocal Raman microscope employing an Ar‐ion laser operating at 532 nm. High‐resolution high‐angle annular dark‐field scanning TEM (HAADF‐STEM) images were recorded by JEM ARM200F Spherical Aberration Corrected TEM. After the sample was dispersed with ethanol, it was dropped on the ultrathin carbon film to dry, and then the sample was tested. The HAADF‐STEM images were processed by the Bandpass filter in the DigitalMicrograph software.

### Synthesis of (NH_4_)_3_[AlMo_6_O_24_H_6_]·7H_2_O Precursor

The (NH_4_)_3_[AlMo_6_O_24_H_6_]·7H_2_O (AlMo6) precursor was prepared according to a published procedure.^[^ ^34^ ^]^ (NH_4_)_6_Mo_7_O_24_·4H_2_O (denoted Mo7, 5.19 g, 4.2 mmol) was dissolved in 80 mL deionized water (DI) water and heated to 100 °C. Al(NO_3_)_3_·9H_2_O (1.84 g, 4.8 mmol) was dissolved in 20 mL DI water, and slowly added into the above solution with constant stirring. The pH of the mixed solution was kept at around 3. As the solution continued to evaporate, a white crystalline precipitate was formed. After 1 h, the product was isolated via filtration, washed by deionized water, and then dried at room temperature. IR (ATR, major absorbances, cm^−1^): 1614 (*δ*OH, m), 1415 (*δ*NH, s), 923 (*ν*Mo = O, vs), 879 (*ν*Mo = O, vs), 635 (*ν*Mo—O—Mo, vs).

### Synthesis of AlO@Mo_2_N‐NrGO

The production of PANI/GO suspension was prepared according to the previous work.^[^ ^25^ ^]^ 20 mL GO solution (0.25 mg mL^−1^), prepared by the modified Hummer's method, was formed into homogeneous suspension by ultrasonic treatment during 2 h. Subsequently, 100 mg aniline was dissolved in 10 mL HCl solution (1.0 mol L^−1^) and mixed with the GO solution under stirring. After 30 min, 0.5 mL H_2_O_2_ solution (30%) was added into the above mixture and stirred for 24 h at room temperature without interruption. The as‐prepared green solution was washed to neutral by centrifugation, followed by adding deionized water to prepare a 20 mL PANI/GO solution. Subsequently, 54 mg AlMo6 was dissolved in 15 mL DI water, followed by mixing with the PANI/GO suspension to obtain AlMo6‐PANI/GO. Then AlMo6‐PANI/GO precursor powder can be obtained by the freeze‐drying. Finally, the obtained product (50 mg) was grinded with urea (40 mg) and thermal treated in H_2_/Ar atmosphere at 700 °C for 3 h to produce Al@Mo_2_N‐NrGO. In addition, various temperatures of 600, 700, and 800 °C were used to control the phase transformation.

### Synthesis of Mo_2_N‐NrGO and NrGO

The preparation of Mo_2_N‐NrGO and NrGO was similar to the preparation of AlO@Mo_2_N‐NrGO with a few differences. Mo_2_N‐NrGO was prepared using Mo7 as precursors instead of AlMo6, while NrGO was prepared without using any POMs.

### Electrochemical Measurements

HER performances of AlO@Mo_2_N‐NrGO, Mo_2_N‐NrGO, and NrGO catalysts were tested on a CHI760E electrochemical workstation (CH Instruments, China) by a three‐electrode configuration in 1.0 m KOH aqueous solution at room temperature. The above catalysts were coated on 1.0 cm^2^ carbon paper (CP) with a loading of 0.5 mg cm^−2^ as the working electrode. Reversible hydrogen electrode (RHE) and a graphite rod were as the reference and counter electrodes, respectively. The 20% Pt/C on CP were measured as a comparison. Linear sweep voltammetries (LSV) were tested in 1.0 m KOH from 0.05 to −0.3 V at a scan rate of 5 mV s^−1^. In addition, 95% iR compensation was applied for above catalysts. Cyclic voltammetry (CV) was obtained from 0.1 to 0.2 V (vs RHE) with sweep rates of 20, 40, 60, 80, 100 mV s^−1^. Electrochemical impedance spectroscopy (EIS) was measured at open‐circuit voltage and various overpotentials with frequencies from 0.1 to 10 000 Hz with an AC voltage of 5 mV. The electrochemical stability test was obtained using the *i*–*t* plot.

The TOF of the AlO@Mo_2_N‐NrGO catalyst is calculated according to the following equation

(1) $$\mathrm{TOF}=I/\left(2F\times n\right)$$

where *I* represent the measures current during LSV, *F* is the faraday constant (96 485 C mol^−1^), and *n* is the mole amount of active Mo site calculated by *n* = m × wt%/95.94 (m is loading mass of AlO@Mo_2_N‐NrGO on carbon paper, wt% is mass percentage of Mo atoms obtained by ICP‐AES elemental analysis, as shown in Table S4, Supporting Information).

To test the Faradaic Efficiency (FE), the H_2_ products are collected over water by a pneumatic trough. The theoretical H_2_ generation value is calculated using Faraday's law assuming an FE of 100%, based on the following equation

(2) $$H_{2}\;\mathrm{volume}=Q\times V_{m}/\left(2\times F\right)$$

where *Q* is quantity of electricity based on equation:$Q=\int_{0}^{t}i\left(t\right)dt$ from the experimentally determined *i*–*t* plot, *V*_m_ is the gas molar volume at 25 °C, 1.01 × 10^5^ Pa, and *F* is the faraday constant. Analysis of the FE determined the amount of H_2_ generated using AlO@Mo_2_N‐NrGO to be consistent with its theoretical value, with an average FE of ≈99.78%.

### DFT Calculations

The spin‐polarized first‐principles calculations were carried out by the periodic DFT method implemented in the DMol3 code.^[^ ^35^ ^]^ The exchange‐correlation energy was determined by the generalized gradient approximation in form of the Perdew–Burke–Ernzerhof functional.^[^ ^36^ ^]^ The Grimme's PBE+D2 method was used to consider the long‐range dispersion interaction.^[^ ^37^ ^]^ Density functional semicore pseudopotential method was employed for the core treatment,^[^ ^38^ ^]^ while the valance electrons were described by the double‐numerical basis with polarization functions (DNP).^[^ ^39^ ^]^ A (8 × 8 × 1) k‐point grid was adopted to sample the Brillouin‐zone. The transition states were searched by the complete Linear Synchronous Transit/Quadratic Synchronous Transit method and further confirmed by the frequency calculation. The convergence tolerances were set as 1 × 10^−5^ Ha, 2 × 10^−3^ Ha Å^−1^, and 5 × 10^−3^ Å, for energy, gradient, and displacement.

The Mo_2_N (111) surface was modeled using a (4 × 4) unit cell with a four‐layer slab and a 20 Å vacuum region. The atoms in the two bottom layers were fixed, while the other atoms were fully relaxed. To consider the effect of the oxygen doping, two O atoms were introduced onto the Mo_2_N(111) surface to form the O@Mo_2_N model. The AlO@Mo_2_N model was built by (H_2_O)_3_Al(OH) anchoring at the two adjacent oxygen sites of O@Mo_2_N, forming six coordinated Al complex ((H_2_O)_3_AlO_2_(OH)) on Mo_2_N.

The change of free energy (Δ*G*) for an elementary reaction was determined by: Δ*G* = Δ*E* + Δ*ZPE* − TΔS, where Δ*E*, Δ*ZPE*, and Δ*S* is the change of electronic energy, zero‐point energy, and entropy at temperature *T* = 298 K, respectively.

### XAS Measurements

The X‐ray absorption structure spectra Mo K‐edge were collected at BL17B beamline of National Facility for Protein Science (NFPS), Shanghai Synchrotron Radiation Facility (SSRF) Shanghai, China The data and the corresponding reference sample were collected in transmission mode. While Al K‐edge were collected at 4B7A beamline of Beijing Synchrotron Radiation Facility (BSRF). The data were collected in Total Electron Yield (TEY) mode. The sample were grinded and uniformly daubed on the special adhesive tape.

### XAS Analysis and Results

The acquired XAS data were processed according to the standard procedures using the ATHENA module of Demeter software packages. The EXAFS spectra were obtained by subtracting the postedge background from the overall absorption and then normalizing with respect to the edge‐jump step. Subsequently, the *χ*(k) data of were Fourier transformed to real (*R*) space using a hanning windows (d*k* = 1.0 Å^−1^) to separate the EXAFS contributions from different coordination shells.

## Conflict of Interest

The authors declare no conflict of interest.

## Supporting information

Supporting Information

## Data Availability

The data that support the findings of this study are available in the supplementary material of this article.

## References

[1] a) Z. W. Seh , J. Kibsgaard , C. F. Dickens , I. Chorkendorff , J. K. Norskov , T. F. Jaramillo , Science 2017, 355, 146; 10.1126/science.aad4998 28082532

[2] a) Z. Y. Yu , Y. Duan , X. Y. Feng , X. X. Yu , M. R. Gao , S. H. Yu , Adv. Mater. 2021, 33, 2007100; 10.1002/adma.202007100 34117808

[3] a) M. Carmo , D. L. Fritz , J. Mergel , D. Stolten , Int. J. Hydrogen Energy 2013, 38, 4901;

[4] a) J. H. Myung , D. Neagu , D. N. Miller , J. T. Irvine , Nature 2016, 537, 528; 27548878 10.1038/nature19090

[5] a) J. Jin , J. Yin , H. Liu , B. Huang , Y. Hu , H. Zhang , M. Sun , Y. Peng , P. Xi , C. H. Yan , Angew. Chem., Int. Ed. 2021, 60, 14117; 10.1002/anie.202104055 33843135

[6] a) J. Kim , H. Kim , W. J. Lee , B. Ruqia , H. Baik , H. S. Oh , S. M. Paek , H. K. Lim , C. H. Choi , S. I. Choi , J. Am. Chem. Soc. 2019, 141, 18256; 31621315 10.1021/jacs.9b09229

[7] a) L. Gao , Z. Yang , T. Sun , X. Tan , W. Lai , M. Li , J. Kim , Y. F. Lu , S. I. Choi , W. Zhang , C. Ma , S. C. Smith , Y. G. Zhou , H. Huang , Adv. Energy Mater. 2022, 12, 2103943;

[8] a) H. Wang , J. Li , K. Li , Y. Lin , J. Chen , L. Gao , V. Nicolosi , X. Xiao , J. M. Lee , Chem. Soc. Rev. 2021, 50, 1354; 33295369 10.1039/d0cs00415d

[9] a) J. Yin , J. Jin , H. Lin , Z. Yin , J. Li , M. Lu , L. Guo , P. Xi , Y. Tang , C. H. Yan , Adv. Sci. 2020, 7, 1903070; 10.1002/advs.201903070 PMC7237848 32440471

[10] a) D. Zhao , K. Sun , W. C. Cheong , L. Zheng , C. Zhang , S. Liu , X. Cao , K. Wu , Y. Pan , Z. Zhuang , B. Hu , D. Wang , Q. Peng , C. Chen , Y. Li , Angew. Chem., Int. Ed. 2020, 59, 8982; 10.1002/anie.201908760 31515887

[11] a) J. T. Ren , L. Chen , D. D. Yang , Z. Y. Yuan , Appl. Catal., B 2020, 263, 118352;

[12] a) H. Huang , Y. Zhao , Y. M. Bai , F. M. Li , Y. Zhang , Y. Chen , Adv. Sci. 2020, 7, 2000012; 10.1002/advs.202000012 PMC7201256 32382489

[13] a) B. Gao , X. Li , K. Ding , C. Huang , Q. Li , P. K. Chu , K. Huo , J. Mater. Chem. A 2019, 7, 14;

[14] a) D. A. Papaconstantopoulos , W. E. Pickett , B. M. Klein , L. L. Boyer , Phys. Rev. B 1985, 31, 752; 10.1103/physrevb.31.752 9935816

[15] a) N. Han , P. Liu , J. Jiang , L. Ai , Z. Shao , S. Liu , J. Mater. Chem. A 2018, 6, 19912;

[16] H. Jin , X. Liu , Y. Jiao , A. Vasileff , Y. Zheng , S. Z. Qiao , Nano Energy 2018, 53, 690.

[17] a) H. Jin , Q. Gu , B. Chen , C. Tang , Y. Zheng , H. Zhang , M. Jaroniec , S. Z. Qiao , Chem 2020, 6, 2382;

[18] a) H. Jin , H. Yu , H. Li , K. Davey , T. Song , U. Paik , S. Z. Qiao , Angew. Chem., Int. Ed. 2022, 134, 27; 10.1002/anie.202203850 PMC9322295 35437873

[19] J. Xiong , W. Cai , W. Shi , X. Zhang , J. Li , Z. Yang , L. Feng , H. Cheng , J. Mater. Chem. A 2017, 5, 24193.

[20] a) H. Yan , Y. Xie , Y. Jiao , A. Wu , C. Tian , X. Zhang , L. Wang , H. Fu , Adv. Mater. 2018, 30, 1704156; 10.1002/adma.201704156 29164704

[21] J. Zhang , Y. Huang , G. Li , Y. Wei , Coord. Chem. Rev. 2019, 378, 395.

[22] a) Y. Zhang , J. Liu , S. L. Li , Z. M. Su , Y. Q. Lan , EnergyChem 2019, 1, 100021;

[23] A. Blazevic , A. Rompel , Coord. Chem. Rev. 2016, 307, 42.

[24] B. Pattengale , Y. Huang , X. Yan , S. Yang , S. Younan , W. Hu , Z. Li , S. Lee , X. Pan , J. Gu , J. Huang , Nat. Commun. 2020, 11, 4114. 32807770 10.1038/s41467-020-17904-z PMC7431582

[25] S. Liang , S. Zhang , Z. Liu , J. Feng , Z. Jiang , M. Shi , L. Chen , T. Wei , Z. J. Fan , Adv. Energy Mater. 2021, 11, 2002600.

[26] a) W. Liu , X. Wang , F. Wang , K. Du , Z. Zhang , Y. Guo , H. Yin , D. Wang , Nat. Commun. 2021, 12, 6776; 34811357 10.1038/s41467-021-27118-6 PMC8608917

[27] M. Huang , A. Adnot , S. Kaliaguine , J. Am. Chem. Soc. 1992, 144, 25.

[28] J. S. Li , Y. Wang , C. H. Liu , S. L. Li , Y. G. Wang , L. Z. Dong , Z. H. Dai , Y. F. Li , Y. Q. Lan , Nat. Commun. 2016, 7, 11204. 27032372 10.1038/ncomms11204 PMC4822009

[29] G. Yang , Y. Jiao , H. Yan , Y. Xie , A. Wu , X. Dong , D. Guo , C. Tian , H. Fu , Adv. Mater. 2020, 32, 2000455. 10.1002/adma.202000455 32173914

[30] Y. Luo , Z. Zhang , M. Chhowalla , B. Liu , Adv. Mater. 2022, 34, 2108133. 10.1002/adma.202108133 34862818

[31] N. Mahmood , Y. Yao , J. W. Zhang , L. Pan , X. Zhang , J. J. Zou , Adv. Sci. 2018, 5, 1700464. 10.1002/advs.201700464 PMC5827647 29610722

[32] a) L. He , W. Zhang , Q. Mo , W. Huang , L. Yang , Q. Gao , Angew. Chem., Int. Ed. 2020, 59, 3544; 10.1002/anie.201914752 31880061

[33] a) O. Shebanova , E. Soignard , P. F. McMillan , High Press Res. 2006, 26, 87;

[34] K. Nomna , T. Takahashi , T. A. Shiral , M. Mima , Polyhedron 1987, 6, 2.

[35] a) S. Grimme , J. Antony , S. Ehrlich , H. Krieg , J. Chem. Phys. 2010, 132, 154104; 20423165 10.1063/1.3382344

[36] J. P. Perdew , K. Burke , M. Ernzerhof , Phys. Rev. Lett. 1999, 77, 18. 10.1103/PhysRevLett.77.3865 10062328

[37] S. Grimme , J. Comput. Chem. 2006, 27, 1787. 16955487 10.1002/jcc.20495

[38] a) Z. Wu , L. Xu , W. Zhang , Y. Ma , Q. Yuan , Y. Jin , J. Yang , W. Huang , J. Catal. 2013, 304, 112;

[39] W. J. Hehre , Acc. Chem. Res. 1976, 9, 399.

