# Supplementary material for: Atomically Interfacial Engineering on Molybdenum Nitride Quantum Dots Decorated N‐doped Graphene for High‐Rate and Stable Alkaline Hydrogen Production
Source: Adv Sci (Weinh). 2022 Oct 26;9(36):2204949. doi: 10.1002/advs.202204949 (PMC9799021; doi:10.1002/advs.202204949)
Supplement: Supplementary file 1 — Supporting Information [file ADVS-9-2204949-s001.pdf]

Supporting Information

Atomically Interfacial Engineering on Molybdenum Nitride Quantum Dots Decorated N-doped Graphene for High-Rate and Stable Alkaline Hydrogen Production

Yichao Huang,[1,] Wenbo Zhou,[1] Weichao Kong, Lulu Chen, Xiaolong Lu, Hanqing Cai, Yongrui Yuan, Lianming Zhao,* Yangyang Jiang, Haitao Li, Limin Wang, Lin Wang, Hang Wang, Jiangwei Zhang,* Jing Gu,* and Zhuangjun Fan**

Prof. Y. C. Huang, W. B. Zhou, W. C. Kong, L. L. Chen, X. L. Lu, H. Q. Cai, Y. R. Yuan, Prof. L. M. Zhao, Y. Y. Jiang, H. T. Li, L. M. Wang, L. Wang, H. Wang, Prof. Z. J. Fan
State Key Laboratory of Heavy Oil Processing, School of Materials Science and Engineering, China University of Petroleum, Qingdao, Shandong, 266580, China
E-mails: yichaoh@upc.edu.cn (Prof. Y. C. Huang); lmzhao@upc.edu.cn (Prof. L. M. Zhao); fanzhj666@163.com (Prof. Z. J. Fan)
Prof. J. W. Zhang
College of Energy Material and Chemistry, College of Chemistry and Chemical Engineering, Inner Mongolia University, Hohhot, 010021, China
E-mail: zjw11@tsinghua.org.cn; jwz@imu.edu.cn
Prof. J. Gu
Department of Chemistry and Biochemistry, San Diego State University, 5500 Campanile Drive, San Diego, California, 92182-1030, USA
E-mail: jgu@sdsu.edu
1 These authors contributed equally to this work.

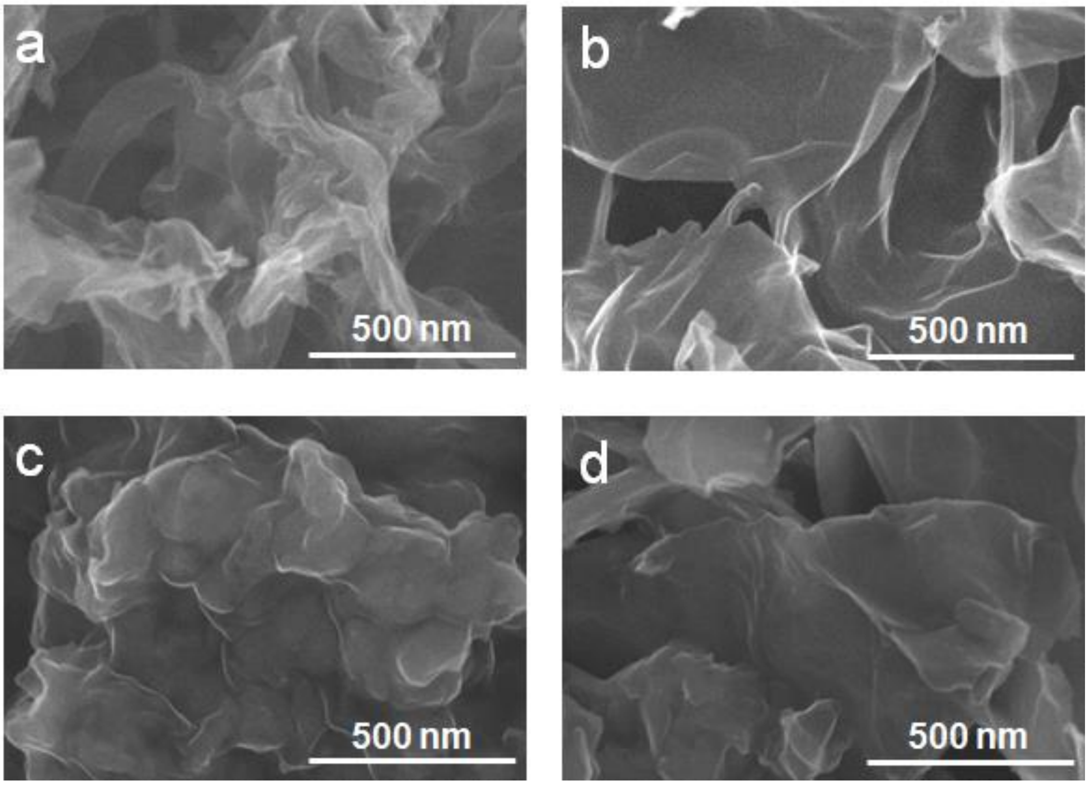

Figure S1. SEM images of **a.** GO, **b.** PANI/GO, and **c.** AlMo6-GO. **d.** AlMo6-PANI/GO nanosheets.

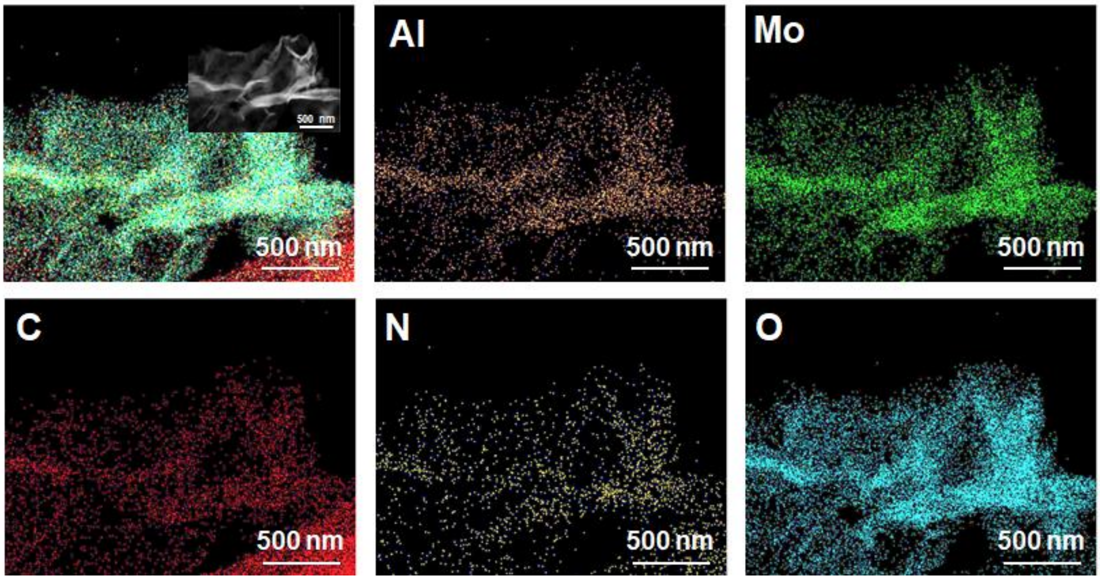

Figure S2. The EDX elemental mappings of Al (orange), Mo (green), C (red), N (yellow) and O (blue) elements in the AlMo6-PANI/GO nanosheets.

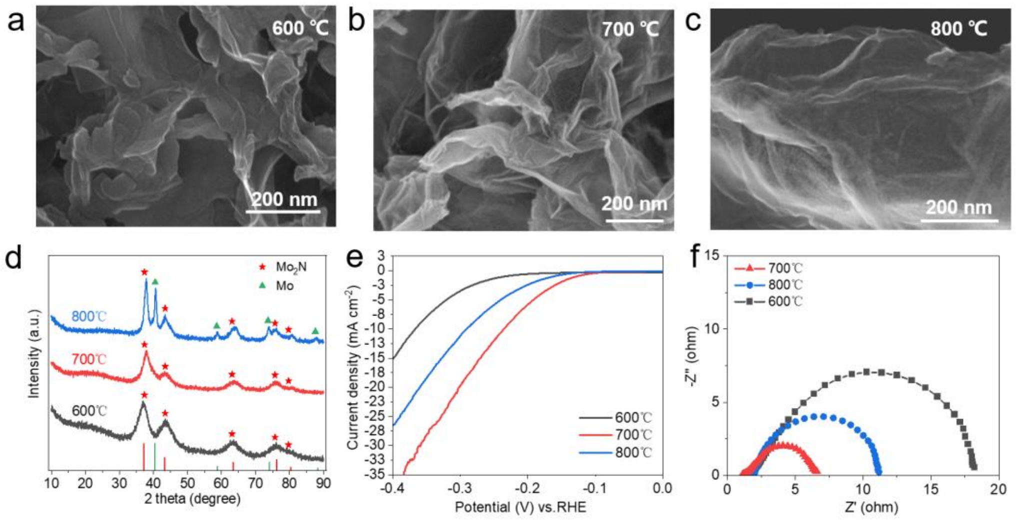

Figure S3. a~c. The SEM of different carbonization temperatures (**a.** 600°C, **b.** 700°C, **c.** 800°C). **d.** The XRD spectra of three samples, 600°C and 700°C correspond to typical Mo_2N phases, while XRD characteristic lines of Mo appear at 800°C. **e.** and **f.** LSV and EIS curves of three samples, it can be seen that 700°C not only has the best HER performance, but also has the smallest resistance.

The nitridation temperature has also been investigated. As shown in Figure S3a-S3c, All the AlO@Mo_2N-NrGO-T samples obtained by different nitridation temperatures (T = 600°C, 700°C and 800°C) exhibit a distinct 2D graphene nanosheet structures. As shown in Figure S3d, the sample obtained by 600°C (AlO@Mo_2N-NrGO-600) has similar Mo_2N phase to AlO@Mo_2N-NrGO-700. However, the AlO@Mo_2N-NrGO-800 shows higher and narrow diffraction peaks, indicating the severe agglomeration of Mo_2N nanoparticles at a higher temperature. Moreover, the AlO@Mo_2N-NrGO-800 shows new peaks of metallic Mo species, which may due to the further reduction of Mo_2N at 800°C by H_2/Ar. Further HER assessments of the temperature-varied samples in 1.0 M KOH electrolyte show that the AlO@Mo_2N-NrGO-700 sample exhibits the best HER activity among all these three samples (Figure S3e). The lower HER activity of AlO@Mo_2N-NrGO-600 may attribute to the lower conductivity, while the lower HER activity of AlO@Mo_2N-NrGO-800 may due to the aggregation and lose of Mo_2N active sites (Figure S3f). Thus, the optimized nitridation temperature is 700 °C and AlO@Mo_2N-NrGO-700 is named AlO@Mo_2N-NrGO for short in this work.

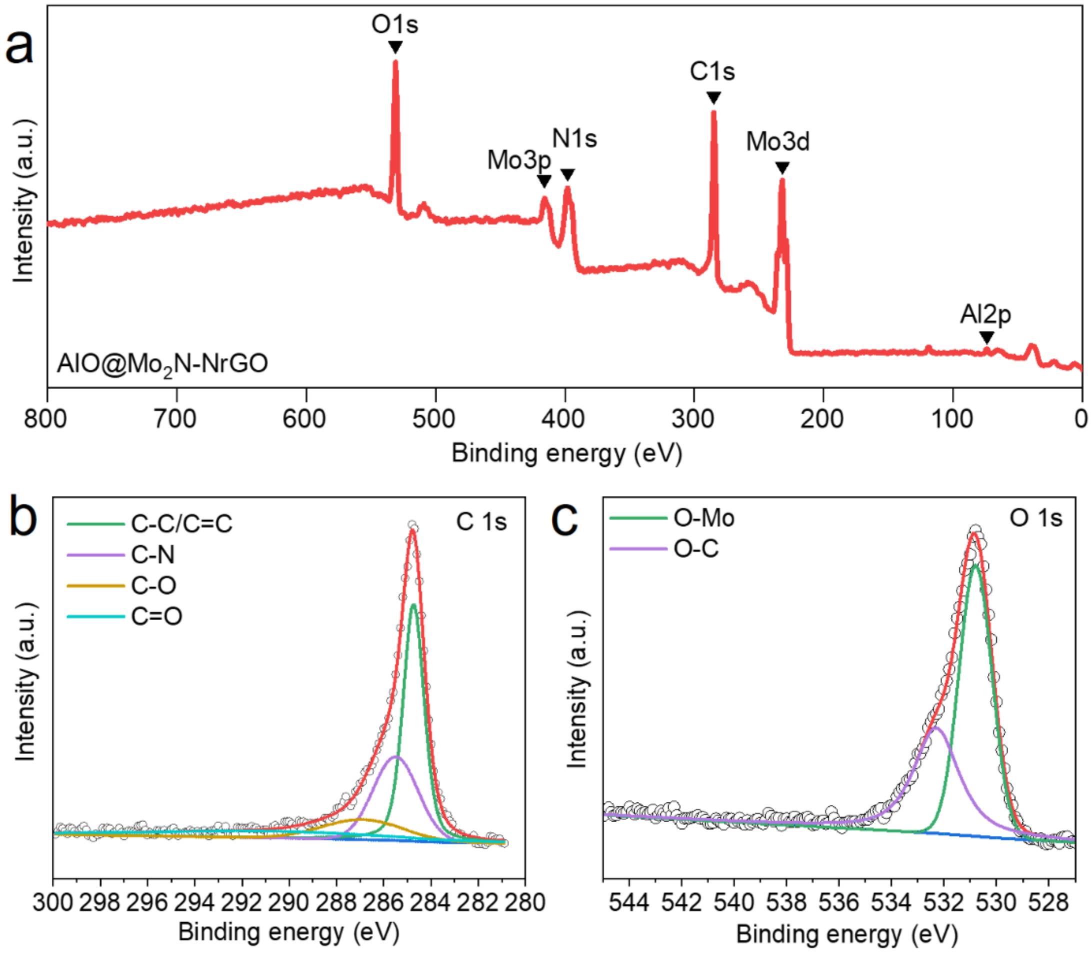

Figure S4. a. XPS survey spectrum of AlO@Mo_2N-NrGO **b-c.** High-resolution XPS signals of **b** C 1s; **c** O 1s.

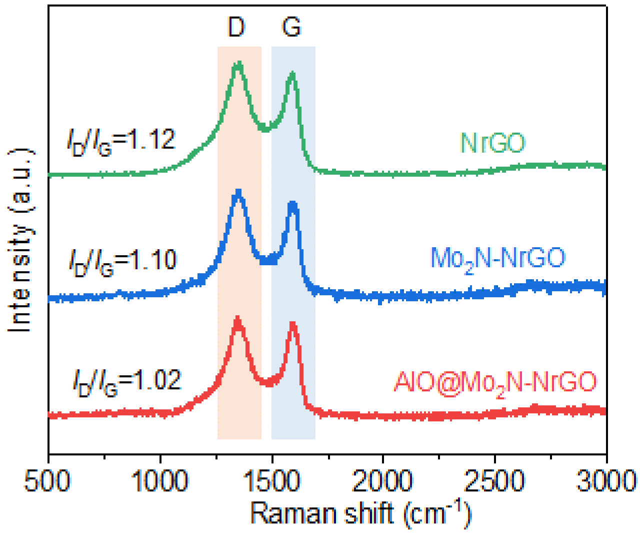

Figure S5. Raman spectra of AlO@Mo_2N-NrGO, Mo_2N-NrGO and NrGO.

The carbon structure in AlO@Mo_2N-NrGO can be verified by Raman spectrum, where the D band (1350 cm^{-1}) and G band (1580 cm^{-1}) provide the evidence of defects and crystallinity of SP^2 carbon structure, respectively. As shown in Figure S5, the intensity ratios (I_D/I_G) of AlO@Mo_2N-NrGO and Mo_2N-NrGO are close to that of NrGO, indicating that the formation of AlO@Mo_2N and Mo_2N quantum dots does not destroy the graphitization of NrGO.

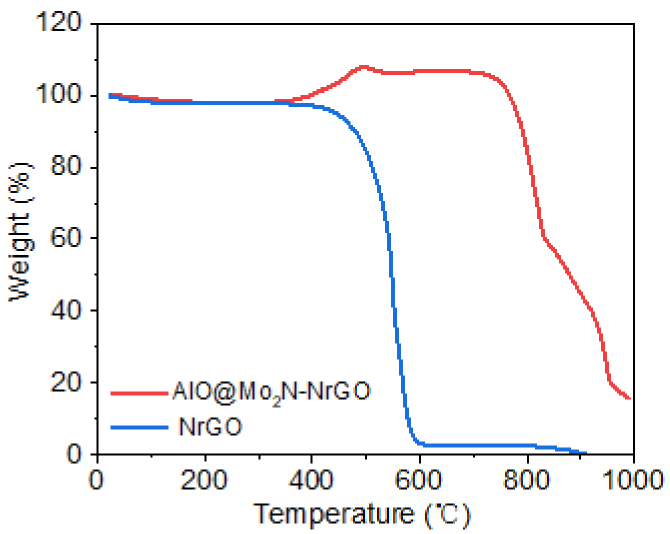

Figure S6. TGA curves of AlO@Mo_2N-NrGO and NrGO. The initial weight gains at 300~500 °C is due to the gradual oxidation of Mo_2N to MoO_3, and then the slow weight at 500~750 °C is due to the combustion of nitrogen-doped graphene (NrGO) and the oxidation of Mo_2N to MoO_3 with remaining weight of ca. 106.5 wt.%. Demonstrating that the NrGO has been completely oxidized and disappeared at 750 °C, the NrGO content is estimated to be ca. 23.8 wt.% according to the following equation: m(NrGO) ≈1-106.5 wt.% * [M(Mo_2N)]/[2*M(MoO_3)] ≈1-106.5 wt.%*205.92/(2*143.94) ≈23.8 wt.%. Therefore, the content of AlO@Mo_2N nanosheet can be estimated to be 76.2 wt.% via the following equation: m(AlO@Mo_2N)=1-m(NrGO). The above results are in accord with elemental analysis results as shown in Table S1.

The presence of carbon matrix can also be verified by thermal gravimetric analysis (TGA) (Figure S6), which is believed to play important roles in prohibiting the aggregation of Mo_2N nanoparticles, stabilizing the nanosheet structures and improving the overall conductivity of AlO@Mo_2N-NrGO electrocatalyst.

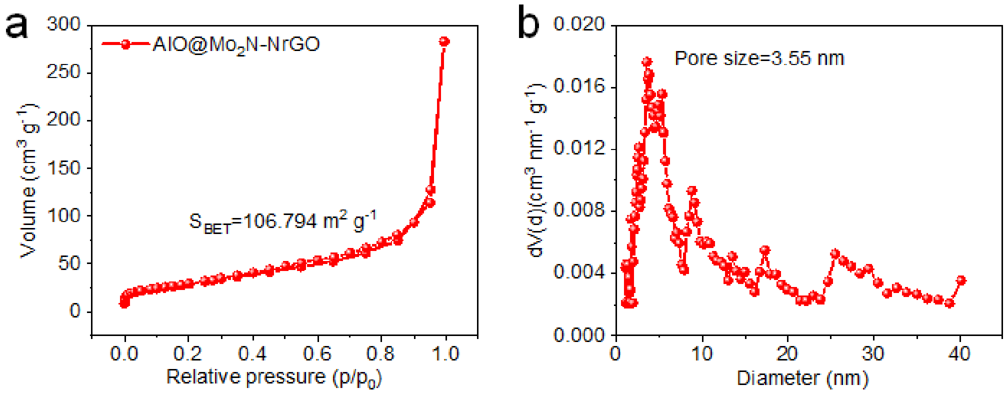

Figure S7. a. N_2 adsorption-desorption isotherm of AlO@Mo_2N-NrGO. **b.** Hole size distribution of AlO@Mo_2N-NrGO.

The porous property of AlO@Mo_2N-NrGO nanosheet is revealed by N_2 adsorption-desorption isotherm measurements (Figure S7a). Meanwhile, the specific surface area and pore size of the AlO@Mo_2N-NrGO are measured by the Brunauer-Emmett-Teller (BET) and the Barrett-Joyner-Hallender methods, exhibiting a high specific BET surface area of 106.79 $m^2 \cdot g^{-1}$ and an average pore size of 3.55 nm (Figure S7b), showing the characteristics of mesoporous structure with an enriched porosity.

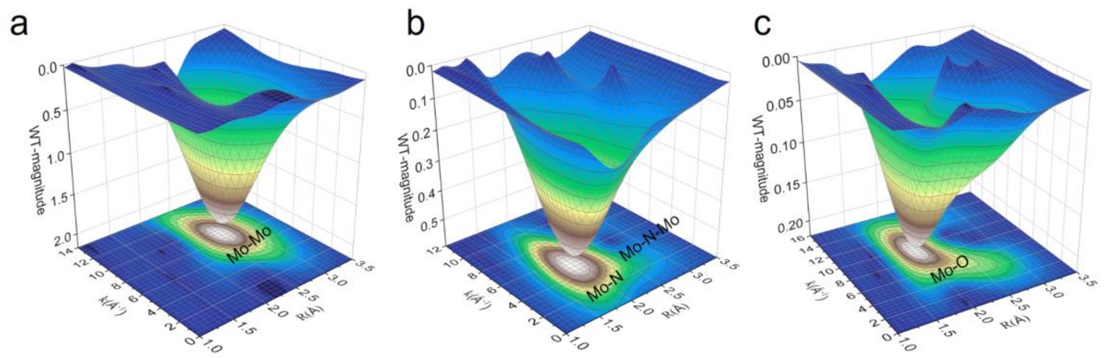

Figure S8. 3D contour Wavelet transform extended X-ray absorption fine structure map with 2D projection of **a.** Mo foil, **b.** Mo_2N and **c.** MoO_3.

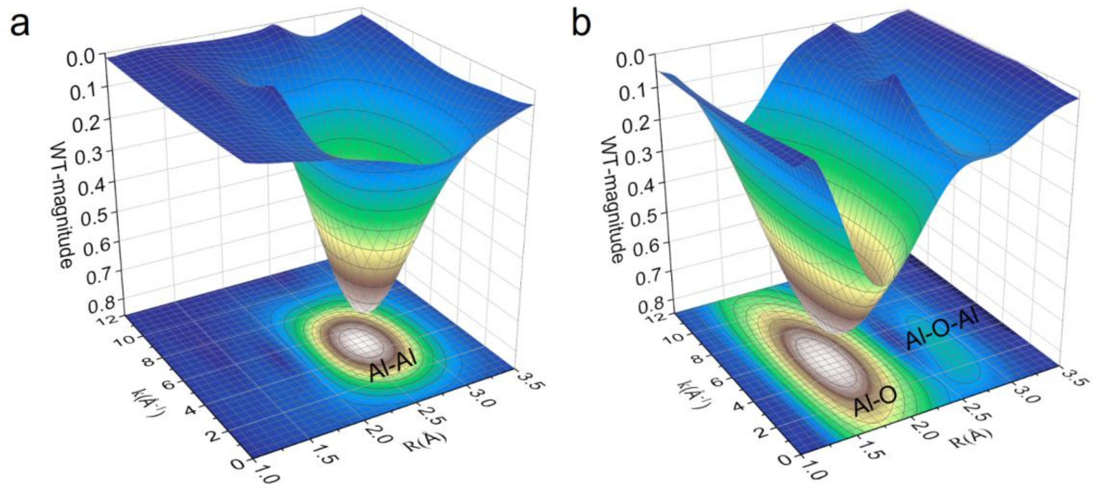

Figure S9. 3D contour Wavelet transform extended X-ray absorption fine structure map with 2D projection of **a.** Al foil and **b.** Al_2O_3.

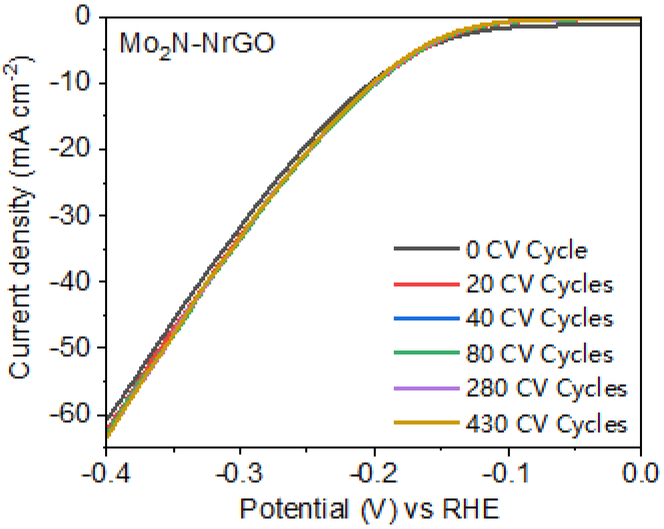

Figure S10. Polarization curves of Mo_2N-NrGO after different CV cycles in 1.0 M KOH with a scan rate of 5 mV s^{-1} under three-electrode configuration.

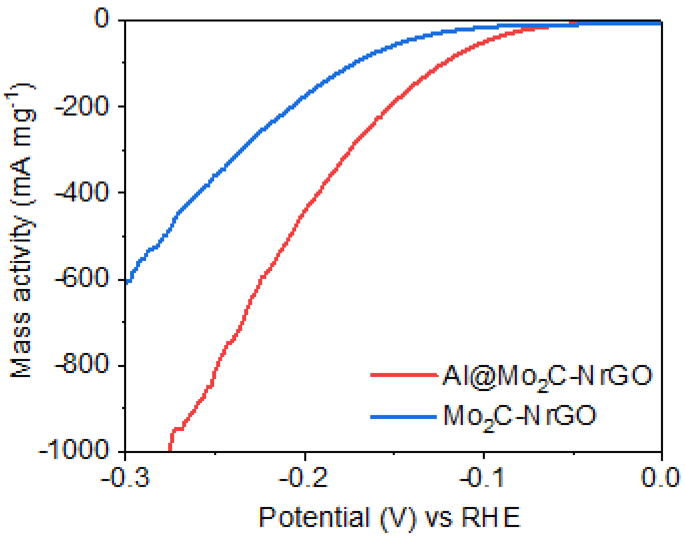

Figure S11. The mass activity of AlO@Mo_2N-NrGO and Mo_2N-NrGO.

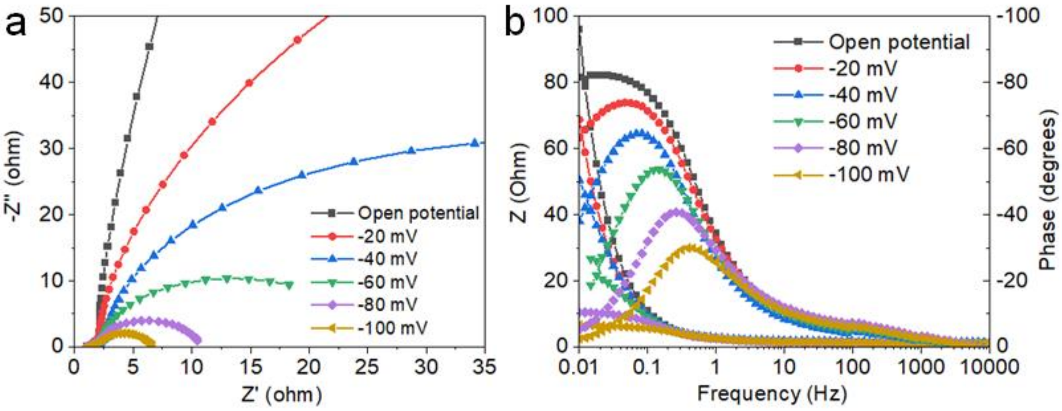

Figure S12. EIS of AlO@Mo_2N-NrGO at various overpotentials. **a.** Nyquist plots and **b.** Bode plots.

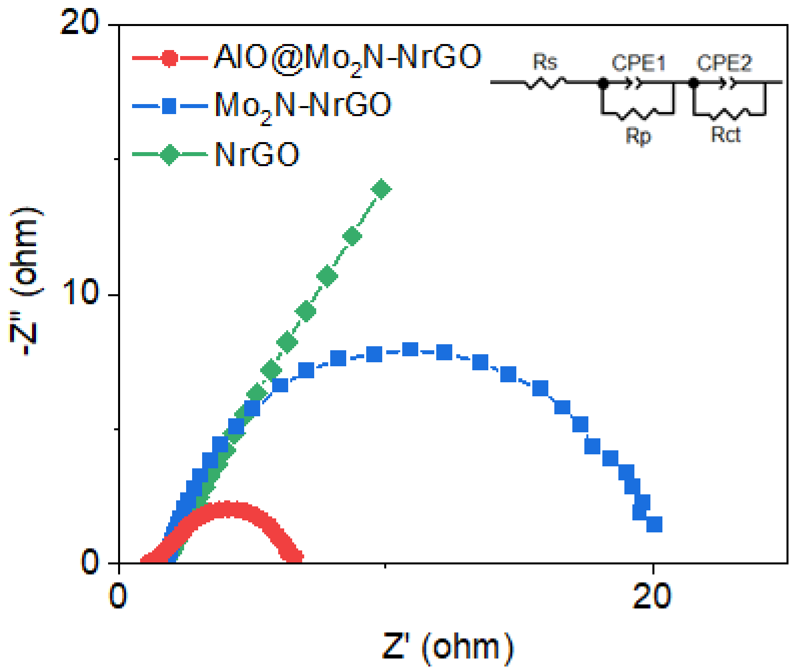

Figure S13. Electrochemical impedance spectroscopy (EIS) of AlO@Mo_2N-NrGO, Mo_2N-NrGO and NrGO. The simplified equivalent circuits are applied to fit the EIS in the inset, where the Rs, Rp and Rct represent the electrolyte, electrode porosity and charge transfer resistance, respectively. CPE is the constant phase angle element, which represents the double layer capacitance of solid electrode in a real-world situation.[1]

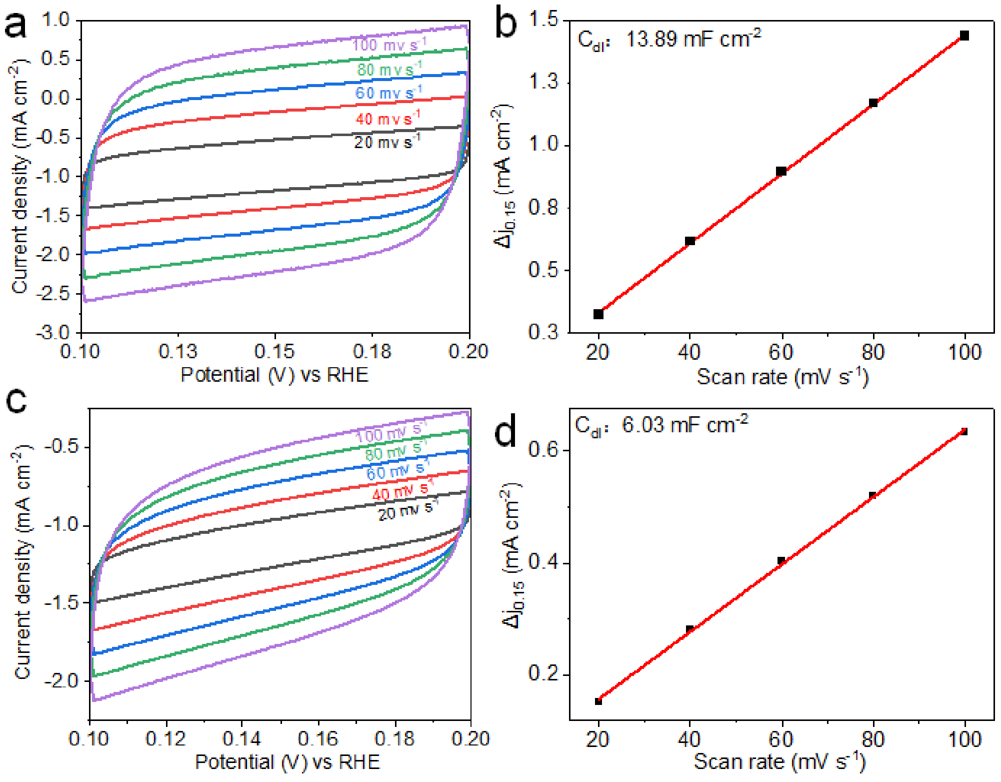

Figure S14. Electrochemical active surface area (ECSA) of **a-b.** Mo_2N-NrGO; **c-d.** NrGO

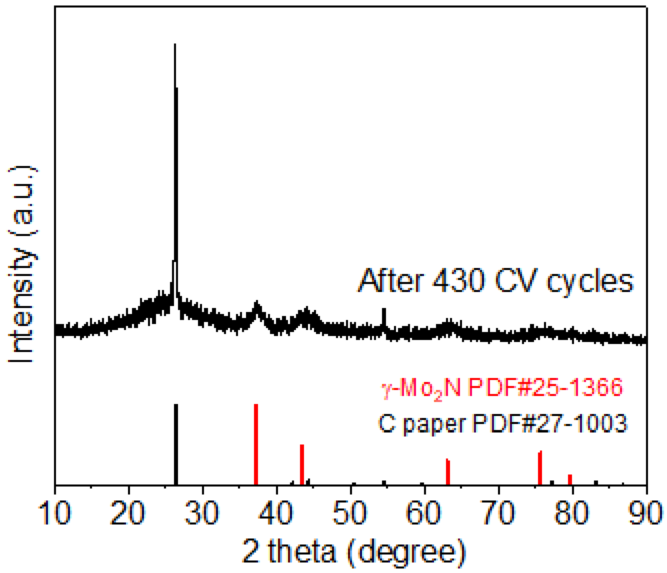

Figure S15. The XRD diagram of AlO@Mo_2N-NrGO after 430 CV cycles.

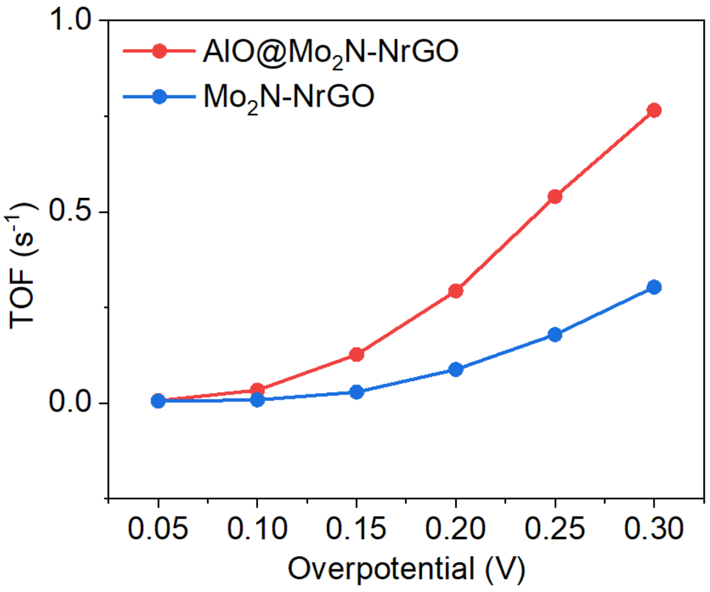

Figure S16. Turnover frequency (TOF) curves of AlO@Mo_2N-NrGO and Mo_2N-NrGO samples.

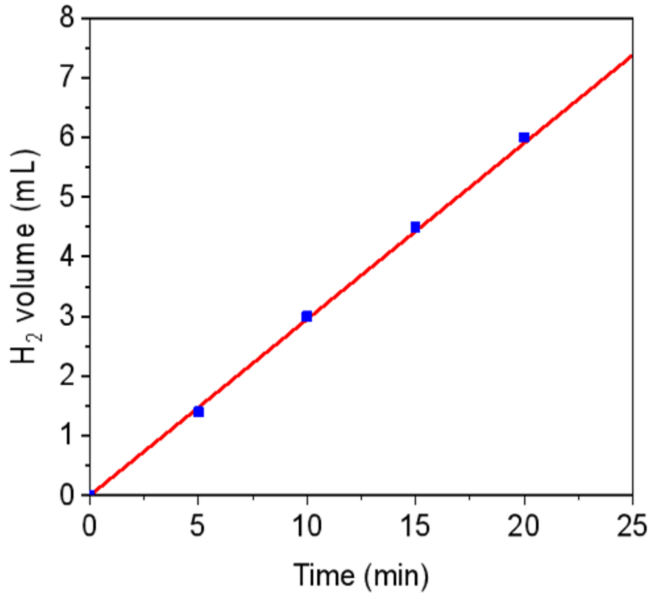

Figure S17. The Faradaic efficient testing of AlO@Mo_2N-NrGO. The amount of H2 theoretically calculated and experimentally measured versus time on the AlO@Mo_2N-NrGO electrocatalyst with a current of 60 mA.

A techno-economic analysis of the AlO@Mo2N-NrGO catalyst

For a single AWE equipment, the hydrogen production cost can be divided into fixed cost and variable cost. The fixed costs include catalyst costs, electrolyzer costs and system auxiliary costs, while the variable costs include power consumption and water consumption, etc. The equation for calculating the cost of unit hydrogen (COUH) from AWE is as follows:

$$\mathrm{COUH} = \frac{\text{Fixed costs + electricity costs + water costs}}{\text{capacity (Nm}^3\text{/hour)}\times\text{time}} \quad (1)$$

If there is no other problem from the AWE except for the difference of catalysts, we may consider components costs of AWE electrolyzer are unchanged in the entire equipment system expect for the catalyst costs. Therefore, we may use the following equation to evaluate the COUH of catalyst:

$$\mathrm{COUH}_{cat} = \frac{\text{Catalyst costs + electricity costs + water costs}}{\text{capacity (Nm}^3\text{/hour)}\times\text{time}} \quad (2)$$

Herein, the COUH of AlO@Mo_2N-NrGO catalyst is compared with that of the state-of-the-art Pt/C (20 wt.%) catalyst to see the economic value it can create. The catalyst costs in comparison of Pt/C (20 wt. %) and AlO@Mo_2N-NrGO is listed in Table 1. The cost of AlO@Mo_2N-NrGO catalyst is about 6.12 US dollars per gram based on the costs of raw materials and fabrication, while the cost of Pt/C (20 wt.%) catalyst is 72.45 US dollars per gram. Therefore, the costs of Pt/C (20 wt.%) and AlO@Mo_2N-NrGO catalysts are 0.71 US dollars and 0.06 US dollars, respectively, when the working electrode area is set to 19.6 cm^2 (diameter = 5 cm) and the loading mass is 0.5 mg/cm^2.

Table 1. Catalyst costs in comparison of Pt/C (20 wt. %) and AlO@Mo_2N-NrGO.

Catalysts	Catalysts cost (US dollar)
Pt/C (20 wt. %)	0.71
AlO@Mo_2N-NrGO	0.06

Notes: Only cathodic catalyst is considered to see the economic value AlO@Mo_2N-NrGO can create, comparing to that of commercial Pt/C (20 wt. %). The working electrode area is set to 19.6 cm^2 (diameter = 5 cm) and the loading mass is 0.5 mg/cm^2.

Based on our long-term stability in Figure 4f, the time is set to 300 hours and the cathodic overpotential is set to 300 mV. As shown in Figure 4b and 4f, the current density of AlO@Mo_2N-NrGO catalyst is about 114 $mA\cdot cm^{-2}$ at an overpotential of 300 mV, while the current density of Pt/C (20 wt.%) catalyst is about 78 $mA\cdot cm^{-2}$ at an overpotential of 300 mV. According to the recent reported excellent OER catalyst by Ren et al.,[2] the S-doped Ni/Fe (oxy)hydroxide catalyst requires low overpotentials of ~300 mV and ~270 mV to deliver current densities of 114 $mA\cdot cm^{-2}$ and 78 $mA\cdot cm^{-2}$. Therefore, in this AWE system, AlO@Mo_2N-NrGO or Pt/C (20 wt.%) is applied as a HER catalyst, while the S-doped Ni/Fe (oxy)hydroxide is used as an OER catalyst.

The amount of hydrogen production is related to the current densities (114 $mA\cdot cm^{-2}$ for AlO@Mo_2N-NrGO, 78 $mA\cdot cm^{-2}$ for 20 wt.% Pt/C), working electrode area (19.6 cm^2) and lifetime (300 hours). As shown in Figure 4f, AlO@Mo_2N-NrGO catalyst exhibits an outstanding stability over 300 hours at an overpotential of 300 mV (current density of 114 $mA\cdot cm^{-2}$). We assume that the 20 wt.% Pt/C catalyst can also perform a stability over 300 hours at an overpotential of 300 mV (current density of 78 $mA\cdot cm^{-2}$, see Figure 4b). Thus, the hydrogen capacity of catalyst can be calculated according to equation (3):

Hydrogen Capacity = Current × 3600 × V_m / (10^3× F × 2) (3)

Where the unit of hydrogen capacity is Nm^3/h; F stands for Faradaic efficiency, 96485 C/mol; The unit of current is A; V_m stands for gas molar volume in standard state, 22.4 L/mol.

The electricity costs of catalyst can be calculated according to equation (4):

Electricity costs = Power consumption × electricity price (4)

Where Power consumption = potential × current × time (5)

At present, the average price of industrial electricity in China is 0.11 US dollar/kWh. The electricity costs of these two catalysts are shown in Table 2.

Table 2. Electricity costs of three catalysts.

Catalysts	Potential (V)	Current (A)	Time (hour)	Hydrogen Capacity (Nm^3/h)	Power consumption (kWh)	Electricity costs (US dollar)
Pt/C	1.80	1.53	300	6.39×10^{-4}	0.83	0.09
AlO@Mo_2N-NrGO	1.82	2.23	300	9.34×10^{-4}	1.22	0.13

Notes: the working time is set to 300 hours, while the working electrode area is set to 19.6 cm^2.

Water cost = water consumption × water price (6)

Unit water consumption (m^3) = Hydrogen Capacity × time × 1000 / V_m × 18 / 10^6 (7)

Which n_{H2O} is consumption of H_2O, M_{H2O} is molar mass of H_2O and ρ is density of water in standard state. The current price of industrial water in Qingdao is 0.57 US dollar/m^3, so the water cost of Pt/C and AlO@Mo_2N-NrGO is 8.78×10^{-5} US dollar and 1.28×10^{-4} US dollar, respectively.

As shown in Table 3, the COUH of AWE using commercial Pt/C (20 wt.%) as a HER catalyst is about 4.17 US dollars per Nm^3H_2. Impressively, the COUH of AWE using AlO@Mo_2N-NrGO as a HER catalyst is only 0.68 US dollars per Nm^3H_2, which can serve 3.49 US dollars per Nm^3H_2 compared to commercial Pt/C (20 wt.%) catalyst.

Table 3. The COUH of catalysts.

Catalysts	Catalyst costs (US dollar)	Hydrogen Capacity (Nm^3/hour)	Lifetime (hour)	Electricity costs (US dollar)	Water costs (US dollar)	COUH (US dollar/Nm^3H_2)
Pt/C (20 wt.%)	0.71	6.39×10^{-4}	300	0.09	8.78×10^{-5}	4.17
AlO@Mo_2N-NrGO-NF	0.06	9.34×10^{-4}	300	0.13	1.28×10^{-4}	0.68

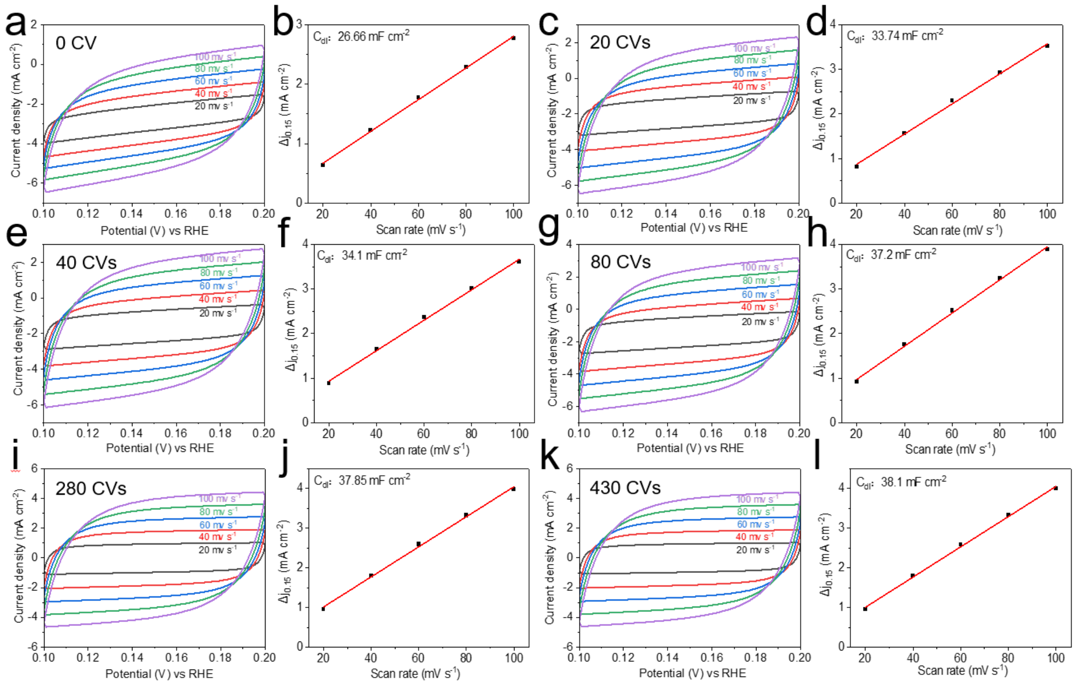

Figure S18. The Cdl of AlO@Mo_2N-NrGO after different CVs from 0 to 430 cycles.

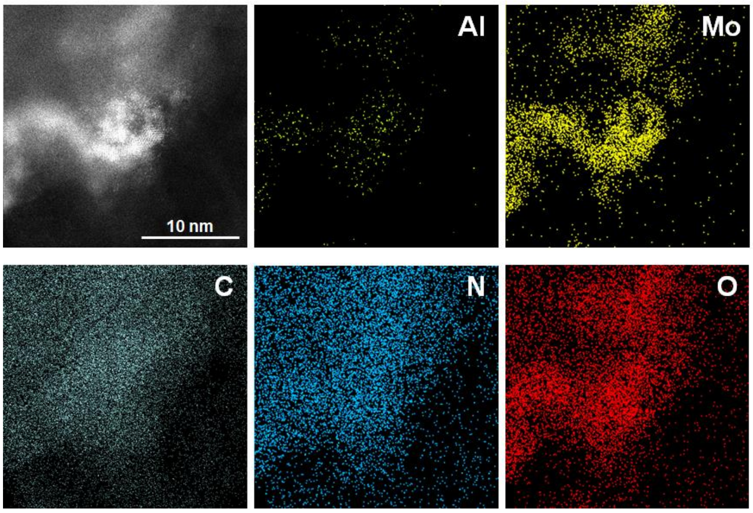

Figure S19. The EDS elemental mappings of high-angle annular dark-field scanning TEM (HAADF-STEM) of Al (green), Mo (yellow), C (cyan-blue), N (blue) and O (red) elements in the A-AlO@Mo_2N-NrGO nanosheets.

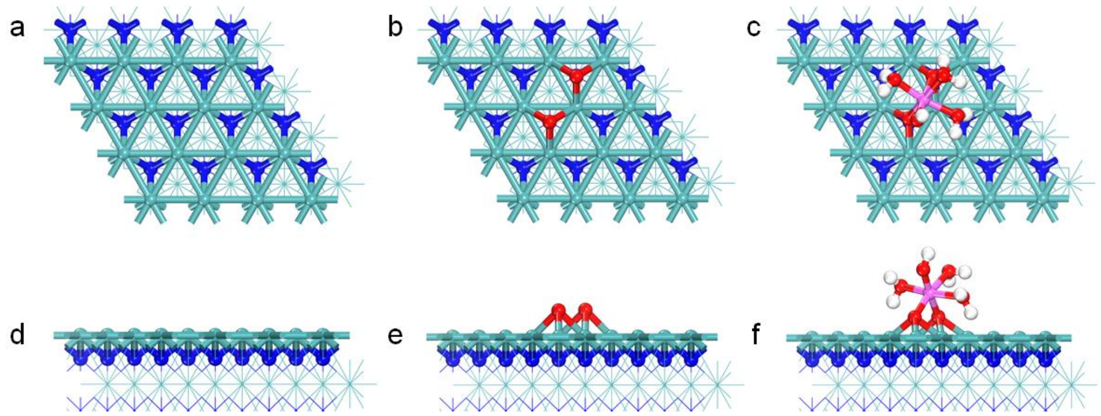

Figure S20. a-c. Top views of Mo_2N, O@Mo_2N and AlO@Mo_2N. **d-f.** Side views of Mo_2N, O@Mo_2N and AlO@Mo_2N. The cyan, blue, red, and white atoms denote Mo, N, O, and H, respectively.

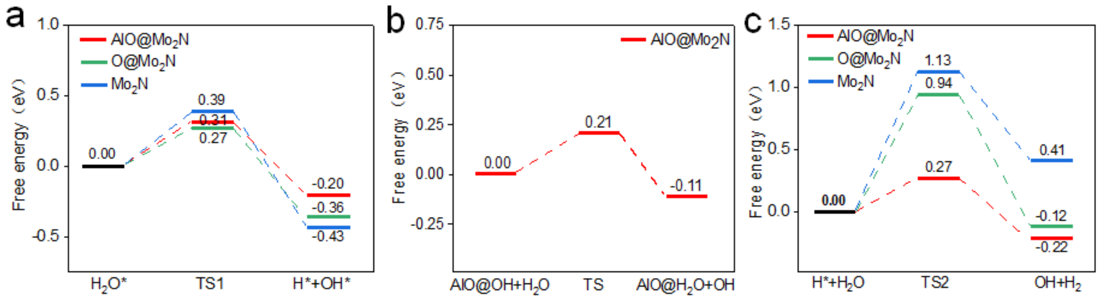

Figure S21. a. Free energy diagram of the Volmer mechanism for HER on AlO@Mo_2N, O@Mo_2N, and Mo_2N. **b.** Free energy diagram for water transfer on AlO@Mo_2N. **c.** Free energy diagram of the Heyrovsky mechanism for HER on AlO@Mo_2N, O@Mo_2N, and Mo_2N. TS is the transition state.

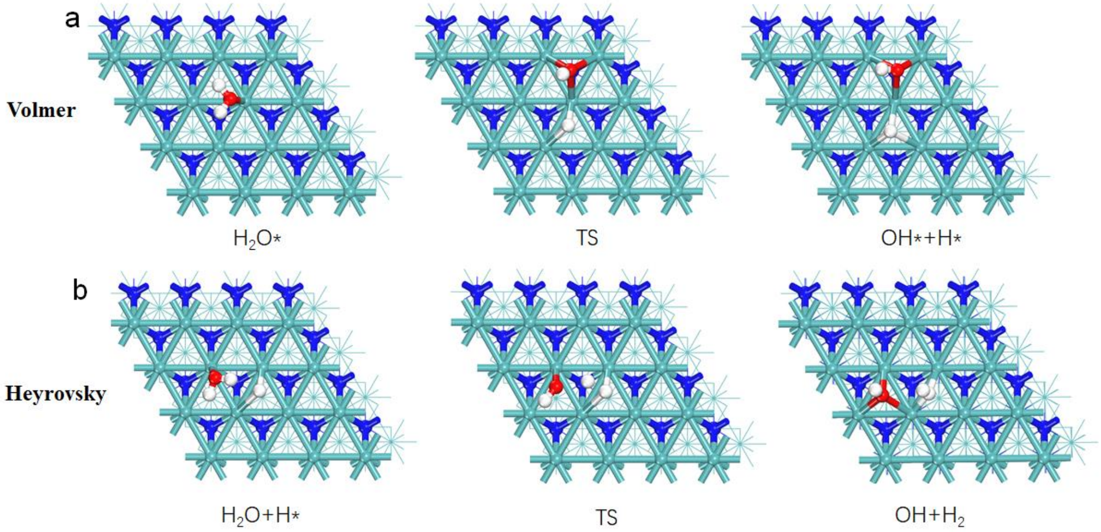

Figure S22. Elementary processes of **a.** the Volmer and **b.** Heyrovsky mechanism for the HER on Mo_2N.

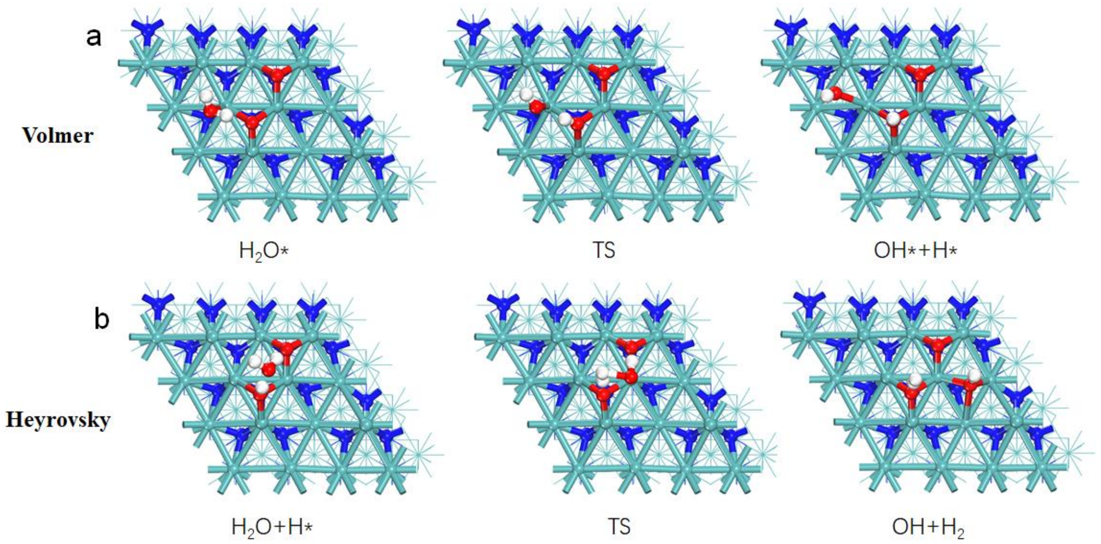

Figure S23. Elementary processes of **a.** the Volmer and **b.** Heyrovsky mechanism for the HER on O@Mo_2N.

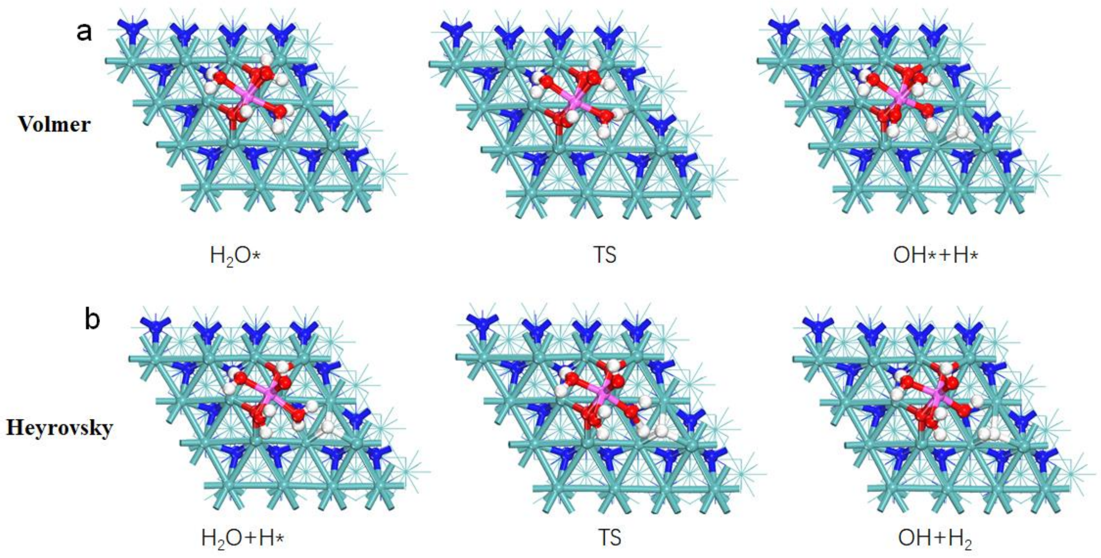

Figure S24. Elementary processes of **a.** the Volmer and **b.** Heyrovsky mechanism for the HER on AlO@Mo_2N.

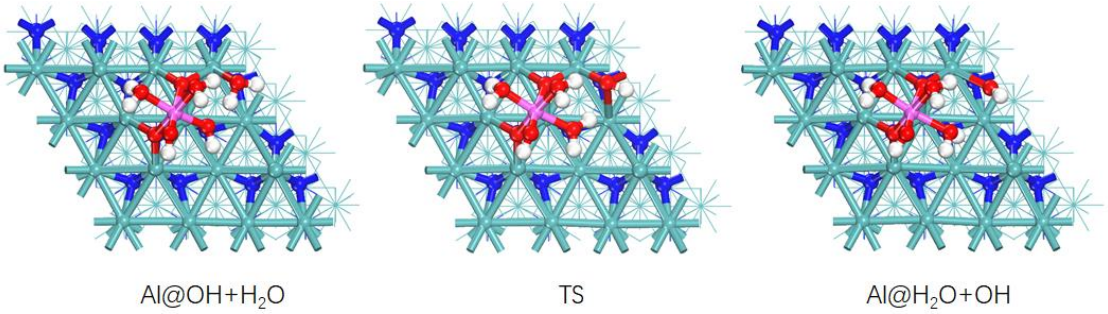

Figure S25. Conversion of $(H_2O)_2AlO_2(OH)_2$ to $(H_2O)_3AlO_2(OH)$.

Table S4. Summary of elemental analysis of AlO@Mo_2N-NrGO electrocatalysts.

Catalysts	Mo content (wt. %) [a)]	Al content (wt. %) [a)]	Al/Mo Atomic ratio [a)]	N content (wt. %) [b)]	N/Mo Atomic ratio [b)]
AlO@Mo_2N-NrGO	53.90	3.62	1:4.20	27.50	1:1.96

Notes: a) Data are determined by ICP-AES elemental analysis; b) Data are determined from EDX spectrum and ICP-AES elemental analysis.

Table S5. Summary of the HER activity of AlO@Mo_2N-NrGO, Mo_2N-NrGO, NrGO and 20% Pt/C on CP in 1 M KOH

Catalysts	η_{onset} (mV)	η_{10} (mV)	η_{300} (mV)	Tafel slope (mV dec^{-1})	R_s [a)] (Ω)	R_p [a)] (Ω)	R_{ct} [a)] (Ω)	CPE1 -T	CPE1 -P	C_{dl} [b)] (mF cm^{-2})	j_0 [c)] (mA cm^{-2})
AlO@Mo_2N-NrGO	57	83	251	69	1.05	0.99	4.54	0.18	0.44	38.10	0.61
Mo_2N-NrGO	68	126	348	90	1.70	6.27	15.32	0.32	0.45	13.89	0.42
NrGO	-	-	-	410	1.80	1.5	173	0.05	0.60	6.03	0.38
20% Pt/C	8	27	277	45			-			-	0.95

a) Data are measured at η = 100 mV. Notes: R_s, R_p and R_{ct} represent the electrolyte, electrode porosity and charge transfer resistance, respectively. CPE is the constant phase angle element, which represents the double layer capacitance of solid electrode in a real-world situation. b) Data are calculated according to the CV results in Figure 4e and Figure S10. c) Exchange current densities (j_0) are obtained from Tafel curves by using the extrapolation method according to the following equation: $\eta = a + b \log j$, where a is the intercept on the y-axis and b is Tafel slope. Exchange current density (j_0) is calculated when η = 0 V.

Table S6. Comparison of HER performance for AlO@Mo_2N-NrGO with other Mo-based HER electrocatalysts (all potentials versus RHE).

Catalyst	Tafel slope (mV dec^{-1})	η_{10}[a)](mV)	References
**AlO@Mo_2N-NrGO**	**64.0**	**83**	**This work**
MoN	77.5	132	[3]
Co/MoN	134.0	52	[3]
Bulk MoN	162.4	251	[4]
Co_3O_4-Mo_2N NFs	167.0	153	[5]
MoN@PC	127.2	393	[6]
CoMoNx-500 NSAs/NF	70.3	160	[7]
CoMoNx-600 NSAs/NF	52.0	161	[7]
Ni/MoN/rNS	93.3	67	[8]
Mo_2N@NC	67.0	133	[9]
Mo_2C-Mo_2N	59.0	222	[10]
Mo_2N-MoS_2	59.7	190	[11]
MoC	73.3	189	[12]
Mo_2C	70.8	201	[12]
Mo_2C/MoC@NC-1-0.5	78.0	161	[12]
N-Mo_2C	64.0	265	[13]
NP-Mo_2C	64.0	210	[13]
NS-Mo_2C	84.2	223	[13]
Mo_2C/C	96.0	254	[6]
Mo_2C@PC	51.0	177	[6]
Zn-MoS_2	97.0	68	[14]
Cu-MoS_2	68.0	160	[14]
Ni-MoS_2	89.0	190	[14]
Co-MoS_2	144.0	240	[14]
Fe-MoS_2	148.0	250	[14]
MoS_2	101.0	180	[14]

MoO_2	86.4	481	[15]
MoO_2	166.0	>500	[16]
MoO_2@C	60.0	220	[17]
MoO_3	325.0	>500	[18]
Co_3O_4/MoO_3	123.0	210	[19]
MoO_3/g-C_3N_4	172.0	470	[19]
Mo-MOFs-T5	61	330.5	[20]
NENU-500	96	245	[21]
UiO-66-NH_2-Mo-5	59	200	[22]
MoS_2/3D-NPC	51	210	[23]
MoCx	59	151	[24]
MOF-$CoSe_2$	42	330	[25]

Notes: a) represents the overpotential (η) at the current density of 10 mA cm^{-2}.

References

Y. Huang, Y. Sun, X. Zheng, T. Aoki, B. Pattengale, J. Huang, X. He, W. Bian, S. Younan, N. Williams, J. Hu, J. Ge, N. Pu, X. Yan, X. Pan, L. Zhang, Y. Wei, J. Gu, *Nat. Commun.* **2019**, *10*, 982.
L. Yu, L. Wu, B. McElhenny, S.W. Song, D. Luo, F. Zhang, Y. Yu, S. Chen, Z. Ren, *Energy Environ. Sci.* **2020**, *13*, 3439.
J. Sun, W. Xu, C. Lv, L. Zhang, M. Shakouri, Y. Peng, Q. Wang, X. Yang, D. Yuan, M. Huang, Y. Hu, D. Yang, L. Zhang, *Appl. Catal. B* **2021**, *286*, 119882.
Y. Zhu, G. Chen, X. Xu, G. Yang, M. Liu, Z. Shao, *ACS Catal.* **2017**, *7*, 3540.
T. T. Wang, P. Y. Wang, W. J. Zang, X. Li, D. Chen, Z. K. Kou, S. C. Mu, J. Wang, *Adv. Funct. Mater.* **2022**, *32*, 2107382.
J. Yang, F. Zhang, X. Wang, D. He, G. Wu, Q. Yang, X. Hong, Y. Wu, Y. Li, *Angew. Chem. Int. Ed.* **2016**, *55*, 12854.
Y. Lu, Z. Li, Y. Xu, L. Tang, S. Xu, D. Li, J. Zhu, D. Jiang, *Chem. Eng. J.* **2021**, *411*, 128433.
Y. Chen, Y. Wang, J. Yu, G. Xiong, H. Niu, Y. Li, D. Sun, X. Zhang, H. Liu, W. Zhou, *Adv. Sci.* **2022**, *9*, 2105869.
C. Wang, X. Lv, P. Zhou, X. Liang, Z. Wang, Y. Liu, P. Wang, Z. Zheng, Y. Dai, Y. Li, M. H. Whangbo, B. Huang, *ACS Appl. Mater. Interfaces* **2020**, *12*, 29153.
Y. Zang, B. Yang, A. Li, C. Liao, G. Chen, M. Liu, X. Liu, R. Ma, N. Zhang, *ACS Appl. Mater. Interfaces* **2021**, *13*, 41573.
K. Ojha, S. Saha, S. Banerjee, A. K. Ganguli, *ACS Appl. Mater. Interfaces* **2017**, *9*, 19455.
H. Q. Chang, G. H. Zhang, K. C. Chou, *Electrochim. Acta* **2021**, *394*, 139119.
D. Wang, T. Liu, J. Wang, Z. Wu, *Carbon* **2018**, *139*, 845.
Y. Shi, Y. Zhou, D. R. Yang, W. X. Xu, C. Wang, F. B. Wang, J. J. Xu, X. H. Xia, H. Y. Chen, *J. Am. Chem. Soc.* **2017**, *139*, 15479.
R. D. Nikam, A. Y. Lu, P. A. Sonawane, U. R. Kumar, K. Yadav, L. J. Li, Y. T. Chen, *ACS Appl. Mater. Interfaces* **2015**, *7*, 23328.
X. Zhang, Z. Du, X. Luo, A. Sun, Z. Wu, D. Wang, *Appl. Surf. Sci.* **2018**, *433*, 723.
G. Yang, Y. Jiao, H. Yan, Y. Xie, A. Wu, X. Dong, D. Guo, C. Tian, H. Fu, *Adv Mater* **2020**, *32*, 2000455.
J. Li, Y. Cheng, J. Zhang, J. Fu, W. Yan, Q. Xu, *ACS Appl. Mater. Interfaces* **2019**, *11*, 27798.
I. Ahmed, R. Biswas, R. A. Patil, K. K. Halder, H. Singh, B. Banerjee, B. Kumar, Y. R. Ma, K. K. Haldar, *ACS Appl. Nano Mater.* **2021**, *4*, 12672.
X. Shi, A. Wu, H. Yan, L. Zhang, C. Tian, L. Wang and H. Fu, *J. Mater. Chem. A* **2018**, *6*, 20100.
J. S. Qin, D. Y. Du, W. Guan, X. J. Bo, Y. F. Li, L. P. Guo, Z. M. Su, Y. Y. Wang, Y. Q. Lan, H. C. Zhou, *J. Am. Chem. Soc.* **2015**, *137*, 7169.
X. Dai, M. Liu, Z. Li, A. Jin, Y. Ma, X. Huang, H. Sun, H. Wang, X. Zhang, *J. Phys. Chem. C* **2016**, *120*, 12539.
Y. Liu, X. Zhou, T. Ding, C. Wang, Q. Yang, *Nanoscale* **2015**, *7*, 18004.

H. B. Wu, B. Y. Xia, L. Yu, X.-Y. Yu, X. W. Lou, *Nat. Commun.* **2015**, *6*, 6512.
J. Lin, J. He, F. Qi, B. Zheng, X. Wang, B. Yu, K. Zhou, W. Zhang, Y. Li, Y. *Chen*, *Electrochim. Acta* **2017**, *247*, 258.
